# To allow or avoid pain during shoulder rehabilitation exercises for patients with chronic rotator cuff tendinopathy-Study protocol for a randomized controlled trial (the PASE trial)

**DOI:** 10.1186/s13063-024-07973-6

**Published:** 2024-02-21

**Authors:** Birgitte Hougs Kjær, Ann M. Cools, Finn E. Johannsen, Jeanette Trøstrup, Theresa Bieler, Volkert Siersma, Peter S. Magnusson

**Affiliations:** 1 Department of Physical and Occupational Therapy, University Hospital Bispebjerg Frederiksberg (BFH), Bispebjerg Bakke 23, 2400 Copenhagen, NV Denmark; 2 Institute of Sports Medicine Copenhagen, Department of Orthopaedic Surgery, University Hospital Bispebjerg Frederiksberg (BFH), Bispebjerg Bakke 23, 2400 Copenhagen, NV Denmark; 3 Department of Rehabilitation Sciences, Faculty of Medicine and Health Sciences, Ghent University, Campus UZ Gent, Corneel Heymanslaan 10, B3, Entrance 46, 9000 Gent, Belgium; 4 The Danish Clinical Quality Program–National Clinical Registries (RKKP), Regionshuset Aarhus, Hedeager 3, 8200 Aarhus N Aarhus, Denmark; 5 Department of Public Health, The Research Unit for General Practice and Section of General Practice, University of Copenhagen, Oster Farimagsgade 5, 1353 Copenhagen K, Denmark; 6 Department of Health and Medical Sciences, Center for Healthy Aging, University of Copenhagen, Copenhagen, Denmark

**Keywords:** Rotator cuff tendinopathy, Shoulder, Exercise, Rehabilitation, Pain, Strength, SPADI

## Abstract

**Background:**

Rotator cuff (RC) tendinopathy is the most reported shoulder disorder in the general population with highest prevalence in overhead athletes and adult working-age population. A growing body of evidence support exercise therapy as an effective intervention, but to date there are no prospective randomized controlled trials addressing pain as an intervention variable.

**Methods:**

A single-site, prospective, pragmatic, assessor-blinded randomized controlled superiority trial. Eighty-four patients aged 18–55 years with chronic (symptom duration over 3 months) RC tendinopathy are randomized 1:1 to receive shoulder exercise during which pain is either allowed or avoided. The intervention period lasts 26 weeks. During that period, participants in both groups are offered 8 individual on-site sessions with an assigned sports physiotherapist. Participants perform home exercises and are provided with a pain and exercise logbook and asked to report completed home-based exercise sessions and reasons for not completing sessions (pain or other reasons). Patients are also asked to report load and the number of sets and repetitions per sets for each exercise session. The logbooks are collected continuously throughout the intervention period. The primary and secondary outcomes are obtained at baseline, 6 weeks, 26 weeks, and 1 year after baseline. The primary outcome is patient-reported pain and disability using the Shoulder PAin and Disability Index (SPADI). Secondary outcomes are patient-reported pain and disability using Disability Arm Shoulder and Hand short-form (Quick DASH), and shoulder pain using Numeric Pain Rating Scale. Objective outcomes are shoulder range of motion, isometric shoulder muscle strength, pain sensitivity, working ability, and structural changes in the supraspinatus tendon and muscle using ultrasound.

**Discussion:**

The results of this study will contribute knowledge about the treatment strategies for patients with RC tendinopathy and help physiotherapists in clinical decision-making. This is the first randomized controlled trial comparing the effects of allowing pain versus avoiding pain during shoulder exercises in patients with chronic RC tendinopathy. If tolerating pain during and after exercise proves to be effective, it will potentially expand our understanding of “exercising into pain” for this patient group, as there is currently no consensus.

**Trial registration:**

ClinicalTrials.gov NCT05124769. Registered on August 11, 2021.

**Supplementary Information:**

The online version contains supplementary material available at 10.1186/s13063-024-07973-6.

## Introduction

### Background and rationale

Shoulder disorders are the third most common musculoskeletal disorder with a lifetime prevalence of up to 70% in the general population [1, 2]. Shoulder conditions frequently persist and recur, with 54% of patients experiencing ongoing symptoms even after 3 years [3]. Rotator cuff (RC) is the most common cause, accounting for up to 80% of all cases of shoulder pain in primary care [4]. The incidence is high in overhead athletes such as tennis or volleyball players because the sports are associated with high forces in the shoulder during serving and smashing [5]. The prevalence has been estimated to be as high as 14% in the general working-age population [6], and about 23% of the working population with shoulder problems are sick listed [7] with a potential individual productivity loss [8, 9].

RC tendinopathy, also labelled as subacromial pain syndrome or RC-related shoulder pain, is pain localized to the proximal lateral aspect of the upper arm originating from the RC and other subacromial structures [10, 11]. Symptoms include weakness and pain during active shoulder elevation and external rotation [12, 13]. Diagnosis can be substantiated based on a pathoanatomic medical model aimed at identifying the pathologic tissues through a number of physical examination tests to increase diagnostic accuracy [14, 15]. Additionally, pain perception is important in diagnosis, as it aims to guide the intensity and progression of treatment intervention [16, 17].

A growing body of evidence support exercise therapy as an effective intervention for reducing pain and disability and improving function in patients with symptomatic RC tendinopathy [18–20]. Guidance in relation to contextual factors and prescription parameters, such as external resistance, training intensity, duration, and frequency as well as exercise setting (home-based or supervised) are summarized in a systematic review [19]. Some of these prescription parameters have been extensively studied and have often yielded conflicting results, while other parameters have been sparsely investigated.

It is well known that loaded tendons regain collagen formation and tensile strength faster than unloaded tendon [21], and that it can take 12 months or longer before it reaches full maturity and strength after an injury [22–24]. The “optimal” magnitude and volume are unknown, although the currently available studies provide some preliminary guidance with respect to the use of loading in exercise programs: progressive heavy strength training appears to yield strength gains, reduce pain, and improve function and quality of life for patients with RC tendinopathy [25–27]. Inducing or allowing pain during the exercise is currently a consensus in the treatment of patellar and Achilles tendinopathy [28, 29] using a pain-monitoring model [30, 31]; however, there is a gap in the literature in this regard, related to shoulder exercises [19].

Exercise programs allowing pain usually include higher load or levels of resistance, which eventually produce greater improvements in pain reduction following a dose–response effect [32]. In addition, painful exercises can serve as a painful conditioning stimulus to initiate conditioned pain modulation response (CPM), which activates decreased pain inhibitory response decreasing pain-related fear [33].

Several studies of patients with shoulder pain did not allow pain during exercise [26, 34–38] and those studies that did allow pain only accepted pain up to 3 on the NPRS [39–44], but its specific effect has not been examined in a randomized controlled trial. Therefore, it is not clear from this data whether pain production or pain avoidance during exercise improve clinical outcomes [19]. No study to date has examined as a primary intervention the influence of pain allowance versus pain avoidance during a shoulder exercise program on patient outcome in terms of pain, function, and sports or occupational-related shoulder disability.

Treatment of RC tendinopathy can include factors other than the tendon pathology itself. The mechanical model of treating tendon injury with exercises is an isolated biomechanical model that does not account for other possible explanatory variables [12, 45]. Yet, some of these variables are challenging to investigate using quantitative research or questionnaires. An inclusive model with four mechanism domains of exercise-related factors has been proposed: tendon structure, neuromuscular function, psychosocial factors, and pain and sensorimotor processing [46]. This model can improve knowledge of mechanisms applicable to RC tendinopathy and enable development of patient-specific treatment to deliver individualized rehabilitation.

When several effective treatment options with different proposed mechanisms are available (e.g., patient education, load management, sport participation, occupational demands, self-efficacy) and several specific factors are impacted by exercise (tendon structure, neuromuscular function, psychosocial factors, and pain and sensorimotor processing) [46, 47], it is important to try to understand the mechanisms by which treatments work and for whom it may work. This can be done by looking at both the effect mediators and moderators [48, 49]. Treatment effects may be mediated by multiple mechanisms. As an example, patient education (cognitive-behavioral approaches) may reduce pain and disability by decreasing pain catastrophizing and fear avoidance while exercises may work by increasing muscle strength [50].

The RC tendinopathy patient’s perspective on participation in exercise programs has only been sparsely investigated [51]. The importance of understanding the reasons for performing the exercises [44] and being able to manage their own condition and a sense of increased control has been highlighted by patients with RC tendinopathy [51]. Furthermore, a quick and meaningful relief in pain or response to exercise therapy has been mentioned by the patients as a crucial feature of continued engagement with exercise therapy to RC tendinopathy [44]. When this does not happen, the motivation of some patients may decrease [44].

To our knowledge, the patients’ experience, and perspective on allowing pain versus avoiding pain based on tendon loading during an exercise program for RC tendinopathy has not been previously investigated.

### Objectives

The primary purpose of this project is to examine the effect of allowing pain versus avoiding pain during shoulder exercises for patients with symptomatic chronic RC tendinopathy measured on patient-reported pain and disability and objective outcomes. We hypothesize that allowing pain during exercises will result in a better outcome measured on Shoulder Pain And Disability Index (SPADI) (the primary outcome) compared to avoiding pain in patients with RC tendinopathy.

A secondary purpose is in a comparative qualitative sub-study to investigate how the elements of the interventions are perceived and experienced as meaningful for the patients during and after training. Furthermore, to uncover motivating factors and barriers to training and how the value of training can be transferred to everyday and working life. The study acronym is the #PASETrial: Pain during Shoulder Exercise.

### Trial design

**Fig. 1:**
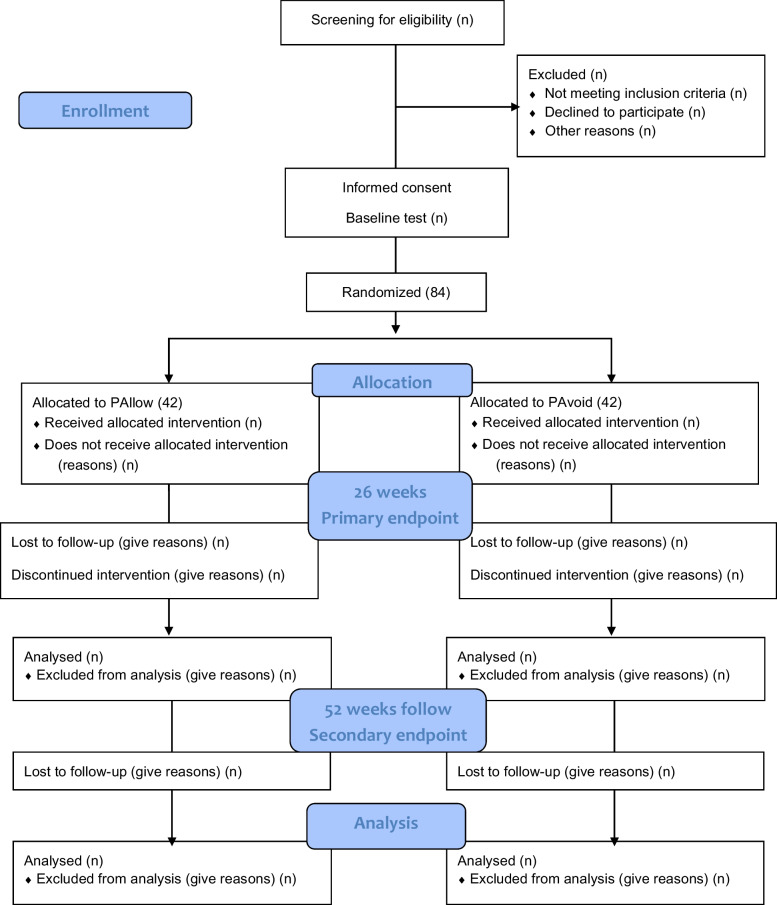
Expected flow of participants through the study. PAllow, group allowing pain during exercise; PAvoid, group avoiding pain during exercise

**Fig. 2:**
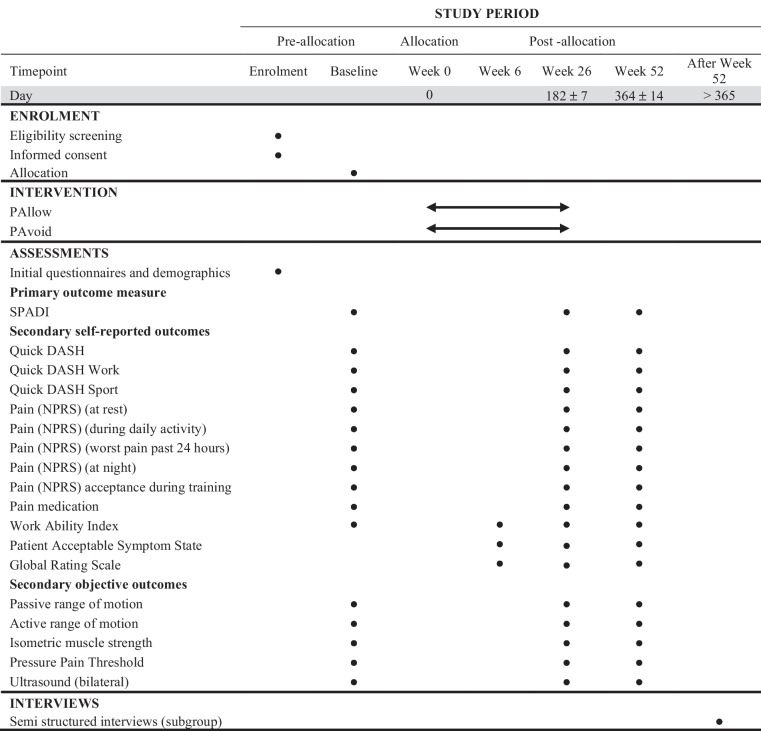
SPIRIT diagram for trial stages of enrolment, interventions, assessments, and visits for participants. SPADI, shoulder pain and disability index; Quick DASH, Disabilities arm, shoulder and hand questionnaire, short form; NPRS, numeric pain rating scale

This is a single-site, prospective, outcome assessor-blinded, pragmatic, randomized, controlled, superiority trial with a two-group parallel design, comparing a pain allowance program (PAllow) with a pain avoidance program (PAvoid). Patients are randomized equally (1:1) to receive either PAllow or PAvoid (Fig. 1). The study has two phases; a main trial phase lasting 26 weeks, which corresponds to the planned duration of the individualized rehabilitation program, and a follow-up phase lasting an additional 26 weeks (Fig. 2).

The study’s primary endpoint is 26 weeks after randomization with outcome assessments at the end of the main trial phase. The secondary endpoint is at week 52 after randomization. During the 26-week follow-up period (from week 26 to week 52), no study-related activities will be performed, except for follow-up or resolution to any possible adverse events occurred during the main trial (weeks 1 to 26).

This study protocol is based on the PREPARE Trial guide [52], the SPIRIT checklist [53], and the Trials’ structured study protocol template [54]. The study report will adhere to the CONSORT 2010 guidelines for reporting parallel group randomized trials. The interventions will be reported according to the TIDieR template for intervention description and replication [55] (Supplementary file 1) and the Consensus on Exercise Reporting Template (CERT) [56] (Supplementary file 2).

## Methods: participants, interventions and outcomes

### Study setting

Participant inclusion, study intervention, and baseline and follow-up assessments in this study are performed at the Department of Physical and Occupational Therapy at the University Hospital Copenhagen, Bispebjerg and Frederiksberg. Participants are recruited from two outpatient clinics at the hospital: the Sports Medicine Clinic and the Occupational Medicine Clinic. The principal investigator (PI) is responsible for all practical management and procedures. Once participants are referred by medical doctors (specialists) in the Sports or Occupational Medicine Clinic, all patients undergo a clinical examination and ultrasound assessment by a sports rheumatologist (FJ) to finally include eligible participants.

### Eligibility criteria

Patients with shoulder symptoms lasting for a minimum of 3 months (chronic) [32] will be included based on the following eligibility criteria.

#### Inclusion criteria

Females and males between 18 and 55 years of age fulfilling the following inclusion criteria.

- Clinical diagnosis of RC (supraspinatus and/or infraspinatus) tendinopathy
- Clinical diagnosis verified by ultrasound (US)

We limit to 55 years to avoid degenerative disease in the RC, and we do not exclude patients with partial RC tear.

The Clinical diagnosis of RC tendinopathy is defined as pain in the proximal lateral aspect of the upper arm, reduced strength, especially during shoulder elevation and external rotation, and a painful active range of motion (ROM) [12, 13, 57]. According to the literature a combination of clinical tests is recommended [14]. At least three of the five following clinical tests should be positive:

- Hawkins [58]
- Jobe/ Empty can [59]
- Neer [60]
- Painful arc (any pain during active elevation) [61]
- Resisted isometric external rotation [62] (pain/ weakness)

The clinical diagnosis is verified by US to further increase the diagnostic precision and to exclude total RC tears, excessive bursa inflammation, and massive calcification. US has high specificity in diagnosing changes in relation to tendinopathy [63, 64] and high reliability when performed using a standardized protocol [65].

#### Exclusion criteria

Patients are excluded if they present with resting pain above 4/10 Numeric Pain Rating Scale (NPRS) and < 90° active elevation of the arm and have had a corticosteroid injection within the previous 12 weeks. Also, patients with isolated subscapularis tendinopathy, total RC tear, acromioclavicular (AC) joint pathology, labrum pathology, glenohumeral joint instability, prior shoulder surgery in any shoulder joint (sterno-clavicular, AC, glenohumeral, scapula-thoracic), glenohumeral osteoarthrosis evaluated on X-ray, rheumatoid arthritis, or periarthrosis are excluded. General exclusion criteria include inability to speak or read Danish, inability to perform physical training, or other conditions negatively influencing compliance.

The following tests are performed to exclude patients with differential diagnoses:

AC-joint pathology:

- Direct AC-palpation pain and Cross over test

Labrum pathology or glenohumeral joint instability:

- Apprehension (instability symptoms and pain) [66]
- and Relocation test
- O’Brien [67]

Rotator cuff tear:

- Drop arm test [14] and
- Resisted isometric external rotation [62] (pain and/or weakness)

In the comparative qualitative sub-study, we will select and invite a sample of 12–16 participants to take part in semi-structured interviews [68] after the 1-year follow-up. This is to get in-depth knowledge of the patients’ experience with the PASE interventions focusing on the PAllow program. To achieve maximum variety, the participants are selected strategically, whereby we obtain a broad group in relation to age, sex, sports participation, and shoulder demands at work.

### Who will take informed consent?

The PI is notified of eligible participants and will then give them detailed oral and written information about the purpose of this study, the study process, and potential risks and benefits. Patient information material, the informed consent form, and a leaflet about patients’ rights in research projects are delivered to the patient prior to the physical baseline testing to give the patients time to read, understand, and carefully consider questions they want answered before giving consent to participate. After written consent to participate, the patients proceed to the scheduled baseline testing including questionnaires.

### Additional consent provisions for collection and use of participant data and biological specimens

No ancillary studies are planned.

## Interventions

### Explanation for the choice of comparators

For both groups the intervention period lasts 26 weeks. During that period, participants in both groups are offered 8 individual on-site sessions with an assigned sports physiotherapist. Additionally, participants have a home exercise program. Two exercise programs were developed with the principal difference being allowing or avoiding pain during the exercises [69] (Table 1).

**Table 1:** Overview PASE interventions

PAllow	PAvoid
Both groups receive 8 individual on-site supervised physiotherapy sessions scheduled in weeks 0, 2, 4, 6, 9, 12, 16, and 20 supplemented with daily home exercises	
Pain NPRS 3–5 allowed during and after exercise	Pain NPRS ≤ 2 accepted during and after exercise
Weeks 0–26: Exercises **F–K** Determined to have a considerable supraspinatus and infraspinatus tendon load with EMG-measured muscle activity above 40% of MVC	Week 0–6: Exercise **A–E** Determined to have a minimal supraspinatus and infraspinatus tendon load with EMG-measured muscle activity below 20% of MVC
**F** External rotation	**A** Sliding
**G** Elevation	**B** Levy
**H** Plyometric	**C** Supine band
**IJ** Eccentric	**D** Extension/ rowing
**K** Isometric	**E** Level 1
Weeks 7–26: Exercise **F–K**	Week 7–26: Exercise **F–K**

See Supplementary file 3 for the specific exercises A–E. See Supplementary file 4 for the specific exercises F–K

*PAllow* group allowing pain during exercise, *PAvoid* group avoiding pain during exercise, *EMG* electromyography, *MVC* maximal voluntary contraction, *NPRS* numeric pain rating scale

### Intervention description

Theoretically, one may provoke or avoid pain during exercises by changing various contextual factors [69, 70]. When the chosen comparison is pain level during and after exercise, the best way to develop 2 exercise programs in which pain is the intervention variable is to consider the assumed tendon load, combined with pain experience during the exercise. This can be accomplished by varying the tendon load based on selective muscle activation during the exercises. One way to determine tendon load based on muscle contraction is electromyography (EMG), assuming that a higher EMG activity during an exercise increases tendon load [69] (Table 1).

#### Training parameters in both groups

The PASE program was developed with special emphasis on patient responsibility and self-management. Thus, part of the intervention in both groups is that participants are offered patient education including load management, advice to gain an understanding of the relation between the occupational demands/situation, sport/leisure time activities, and shoulder training including skills to perform specific shoulder exercises [46]. Finally, patients are provided with knowledge on how to act if shoulder pain occurs during home exercise and activities of daily living. In this way, exercise progression is individualized and guided by the patients’ acceptable symptom response using predefined cutoffs regarding progression in relation to pain according to group allocation.

Initially, the physiotherapist determines the level of each exercise, based on quality assessment of the current individual shoulder function. Patients are instructed and supervised in how to perform exercises with good quality and when to progress, adapt, and act in relation to their current shoulder function. Below we describe the principle for both the PAllow and PAvoid.

For both programs, depending on tissue irritability (expressed in pain) and other factors such as ROM, the exercises may be performed in an isometric way, or dynamic (external resistance or isokinetic) [19]. In general, exercises including external rotation activates the supraspinatus and infraspinatus which has shown to increase the subacromial space [71–73] and therefore favorable.

#### PAllow

**Fig. 3:**
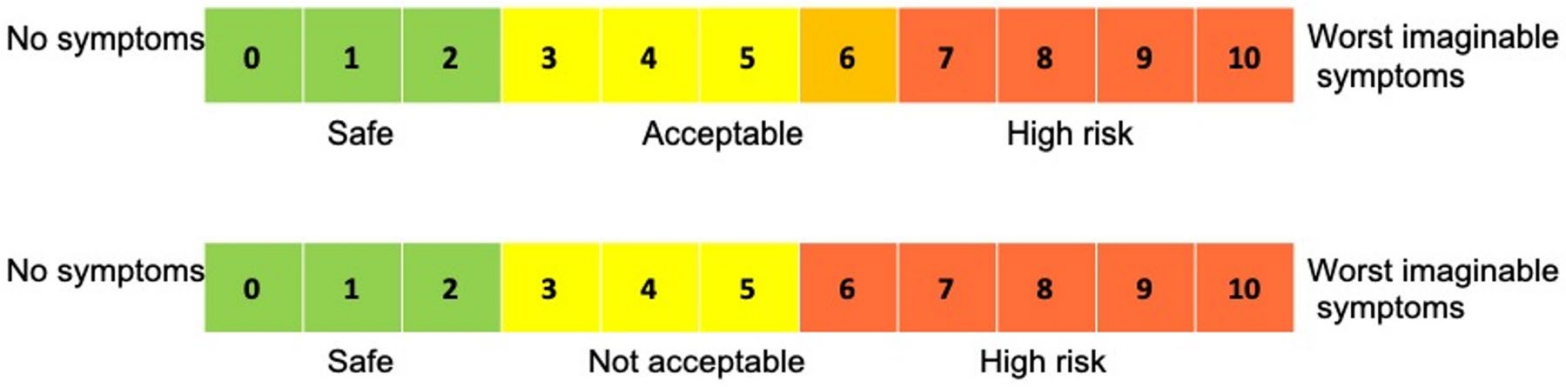
Symptom scale for PAllow (upper scale) and PAvoid (lower scale)

In the PAllow program the selected exercises are considered to have a considerable supraspinatus and infraspinatus tendon load with EMG muscle activity of above 40% of MVC (maximal voluntary contraction) [69, 74]. Management of shoulder pain and/or symptoms during exercises is performed with the use of a symptom scale ranging from 0 to 10, with 10 being worst imaginable symptoms (Fig. 3). The cutoff regarding progression in relation to pain perception during and after exercises is up to 5/10, monitored by NPRS [28, 31, 75, 76]. However, if a patient presents with resting pain of 4/10 at inclusion, pain is allowed to increase by 2/10 during and after the exercises (corresponding to the Minimal Clinical Important Difference (MCID) for NPRS) [76, 77]. This is indicated by the orange square in the symptom scale (Fig. 3).

All the loading exercises (exercise F–K) are performed for 26 weeks (Table 1). External rotation; Elevation; Plyometric; Eccentric exercises (exercise F–IJ) in different levels are the main exercises in PAllow and isometric exercise (exercise K) is used in case of tendon and connective tissue irritability (level 1) (Supplementary file 4).

#### PAvoid

In the PAvoid program, the selected exercises have minimal supraspinatus and infraspinatus tendon load with EMG muscle activity below 20% of MVC [69]. The cutoff regarding progression in relation to pain perception during and after exercises are ≤ 2/10, monitored by NPRS (Fig. 3) [28, 31, 75, 76].

These minimal loading exercises (exercise A–E) are performed for 6 weeks (Table 1). Sliding; Levy; Supine band; Rowing (exercise A–D) in different levels are the main exercises in PAvoid and close chain exercise (exercise E) is used in case of tissue irritability (level 1). In the second part of the exercise protocol (weeks 7–26), increased loading exercises (exercise F–K) are applied, however, without pain exceeding NPRS 2 (Supplementary file 3).

#### Progression parameters

Repetitions are set to 3 sets of 15 repetitions. In general, training should be performed twice a day with at least 6 h between 2 sets (morning/ afternoon) (midday/ late night) [78]. Week 0: two exercises (1 exercise in the morning and a different exercise in the evening); week 1: Add 1 exercise = 3 exercises; week 4 add 1 exercise = 4 exercises. Participants never have more than four exercises, but the exercises themselves progress continuously.

To allow for progression, each exercise has between 3 and 18 levels of difficulty. For all exercises several progression types are possible: increasing resistance, changing the plane, ball versus towel, increasing ROM, increasing kinetic chain demands (perform exercise standing on one leg, or squatting). When patients are familiar with the PASE program, patients are encouraged to progress to new levels of exercise by themselves. Exercises are progressed when satisfactory neuromuscular control is obtained, according to the following criteria: exercise performed with good movement quality, resistance, and repetition accomplished, no compensating movement strategies, performed within the accepted pain limit, steady breathing and general good body control, no need for visual, verbal, or tactile feedback.

#### Supervised physiotherapy sessions

The individual supervised physiotherapy sessions are carried out in the Department of Physical and Occupational Therapy. The following elements are incorporated in the supervision of patients to increase compliance and educate patients to self-management of exercises. Patients are taught to differentiate between inflammatory symptoms, and symptoms associated with muscle soreness, and how to act in relation to these symptoms. Patients are informed about the relevance of each exercise and taught when/how to correct their body posture according to prescriptions for satisfactory neuromuscular control.

During the first supervised session, patients are provided with a small leaflet introducing patients to the subsequent treatment and progression parameters with the purpose of increasing home exercise compliance: Management of shoulder symptoms during home exercises; pain monitoring, and progression (number of exercises, load).

#### Exercise equipment

The following exercise equipment is provided for both the supervised training and home training: Thera-Band soft weight ball of 0.5 or 1 kg and Thera-Band exercise bands (color progression), Thera-Band System, The Hygienic Corporation, Akron, OH 44310, USA.

A personal folder with the pain and exercise logbook combined with exercise manual including the specific exercises is delivered to every participant. The logbooks will be returned to the treating physiotherapist at the 6-week, 12-week, and 26-week follow-up appointment and then collected by the PI. The exercise manuals are the layman’s version of Supplementary files 3 and 4.

#### Treating physiotherapists

The treating physiotherapists responsible for delivering the interventions are assigned post graduate sports physiotherapists with more than 10 years’ experience in specialized sports rehabilitation. Delivery of the two intervention treatments is supported by a manual for the physiotherapists to guide the 26 weeks exercises with the total of 8 supervised physiotherapy sessions. The same physiotherapists deliver exercise programs for both groups, and they are instructed to treat all participants with the same degree of rigor, enthusiasm, and optimism. The same physiotherapist will individually supervise the participant through the intervention period, and if needed due to absence, another assigned physiotherapist will take over.

### Criteria for discontinuing or modifying allocated interventions

The exercise load and progression will be continuously adjusted and either increased or decreased according to the patients’ capabilities. In case of tissue irritability with pain above NPRS 5 in the PAllow group, exercise category K/ level 1 (isometric exercises) may be applied. In case of tissue irritability with pain above NPRS 2 in the PAvoid group, exercise category E/ level 1 may be applied (close chain). In case the patient still feels pain during the exercises in that period, the minimal loading exercises (A–E) are continued.

### Strategies to improve adherence to interventions

The pain and exercise logbooks are used to improve adherence, compliance, and attendance to the training intervention within the 26 weeks of the main trial. In the logbooks, the patients are asked to report completed home-based exercise sessions and reasons for noncompleted sessions (pain or other reasons). The patients are also asked to report the number of sets and repetitions per set for every exercise session, and the load during the exercise. The patients are instructed to complete a pain logbook using the symptom scale (Fig. 2). The self-reported pain/ symptoms will be registered in connection with a training session including immediately before, during, immediately after, 1 h after, or the morning after. In the web-based Research Electronic Data Capture (REDCap), a dashboard report will be created for all patients to monitor whether the email questionnaire has been answered and patients get a reminder if they forget. In case participants fail to attend a supervised exercise session, the treating physiotherapists will contact them by phone to inform them about the importance of adherence to the protocol. Further, efforts will be made to make sure that exercise sessions are scheduled as convenient as possible for participants. Every week, the PI will briefly look at the REDCap dashboard to make sure patients are engaged and, if necessary, talk to the intervention physiotherapist or contact the patient by email or phone.

### Relevant concomitant care permitted or prohibited during the trial

Participants can take pain medication during the trial and continue with existing medical treatments as advised by their general practitioner. All concomitant specific shoulder training in the main trial period (weeks 0–26) will be considered as a violation to comply the intervention. Receiving a corticosteroid injection during the main trial period will also be considered as a violation. But participants will remain in the study, and it will not have consequences for care and attention. Thus, a participant that does not comply to the intervention is categorized non-compliant to the protocol constituting the differences between the intention to treat (ITT) and per protocol analyses.

### Provisions for post-trial care

No post-trial care will be provided to the participants. However, participants are not restricted from getting other treatments after the 26-week intervention period.

### Outcomes

The primary and secondary outcomes are obtained at baseline, 6 weeks, 26 weeks, and 1 year after baseline (Fig. 2).

#### Primary outcome

The primary outcome is the 26-week change from baseline in the Shoulder Pain and Disability Index (SPADI) [79]. SPADI is a widely used shoulder-specific patient-reported outcome (PRO) [80], the most commonly used PRO in trials of conservative interventions [19], and it is considered one of the most responsive shoulder PROs [81–84]. A MCID of 10 points has been identified [85, 86]. The SPADI is a questionnaire of pain and function with 13 items divided into two sub-scales: pain (5 items) and disability (8 items). The responses are indicated on a visual analog scale where 0 = no pain/no difficulty and 10 = worst imaginable pain/so difficult it requires help. The items are summed and converted to a total score out of 100 where a high score indicates greater pain and disability in patients with shoulder disorders [85]. The SPADI is easy and fast to complete [84] and, in a recent systematic review, it was highlighted as one of three PROs for patients with rotator cuff disease for which the psychometric properties are supported by most strong or moderate evidence [87].

#### Secondary outcomes

Secondary outcomes include PROs in addition to measures of range of motion, strength, pain pressure threshold, and ultrasound. PROs include assessment of pain, functional activity level, and health-related quality of life (HRQOL), using the Disability Arm Shoulder Hand short form (Quick DASH) [88–90]. The Quick DASH questionnaire is specified to upper extremity disorders and consists of 11 items divided into 6 items on function and 5 on symptoms. The questionnaire score ranges from 0 to 100 where 0 equals no disability and 100 equals the most severe disability. The Danish validated version was found to be a reliable and responsive outcome measure in a variety of Danish-speaking patients with orthopedic upper extremity problems [89].

Using the Numeric Pain Rating Scale (NPRS), the perceived shoulder pain is assessed at rest, during general activity/ function, at night and as worst pain during the past 24 h with the question “How do you perceive your worst/maximum pain during the past 24 h?” [75, 76]. The minimal clinical important difference (MCID) for NPRS is 2 points [76]. Patients are also asked about pain acceptance (using NPRS) with the questions: “How much pain would you accept during daily activities?’ and ‘How much pain would you accept during exercise?”.

The patient acceptable symptom state (PASS) [91] is an absolute cutoff value that can be helpful to interpret the results of a patient-rated outcome at the individual level by using it as a therapeutic benchmark [92–95]. The PASS is defined as the outcome score at which patients find themselves in a satisfactory condition [96]. The PASS threshold is determined by asking patients if they are satisfied with their current health state [95, 97] with the concrete question: “Taking into account all the activities you have during your daily life, your level of pain, and also your functional impairment, do you consider that your current state is satisfactory? Patients respond to this question with “yes” or “no”. One method to derive the PASS for a patient-reported outcome are the 75th percentile approach, which estimates the PASS as the 75th percentile score of patients who considered their health state acceptable and therefore answered “yes” to the question [92, 94–96, 98]. Once the PASS threshold is determined, the percentage of patients who achieve the PASS and consequently perceive theirselves in an acceptable health status can be reported. Several PASS thresholds for outcome measures used in shoulder patients are reported [92, 94, 96] and a number of shoulder studies [99, 100] have used the PASS to evaluate response to treatment.

The Patient Global Rating Scale (GRS) is used to obtain a global/general impression of recovery from baseline to 26 and 52 weeks after baseline with the question: “Compared to when this treatment first started, how would you describe your shoulder this last week?” This is assessed on a 15-point scale where 7 represents vastly worse, 0 represents unchanged, and + 7 represents much better [101, 102].

Active and passive shoulder ROM will be assessed in scapular plane elevation (standing next to a wall) and in external rotation (supine in 90° abduction) [103, 104] using a digital inclinometer (Baseline Evaluation Instruments, model 12–1057 (ProCare)).

Isometric muscle testing is performed sitting on a stool by MVC of 45° shoulder elevation in scapular plane and external shoulder rotation with arm in neutral [103, 105, 106]. Measurements are performed with a dynamometer (Iso Force Control, model EVO2, 10–400 N, Medical Device Solutions AG).

A semi-objective method for quantifying localized pain is measuring pressure pain threshold (PPT) using a handheld pressure algometer (Algometer Type II; Somedic AB, Sollentuna, Sweden) that consists of a strain gauge. A gradually increasing force is applied to the measured region with the subject (patient) signaling when the sensation of pressure becomes painful [68, 107–109]. The PPT is performed locally (close to the painful shoulder) and on the lower extremity as a control for global pressure pain threshold changes on the following standardized anatomic locations: the descending part of the trapezius muscle (unilaterally), the mid-portion of the deltoid muscle (unilaterally), and the tibialis anterior muscle 5 cm distal to the tibial tuberosity (bilaterally).

The four test sites are first located and marked. The subject is lying prone for the trapezius muscle and seated on a plinth the other measurements. The probe (1 cm^2^) is placed perpendicular to the skin. Pressure is applied at a rate of 30 kPa/s, and participants are instructed to indicate when the sensation changes from a sensation of pressure to the sensation of pain. PPT is measured twice at each site, and the average is calculated.

Quantitative and qualitative US evaluation of tendon healing characteristics in the superior rotator cuff and subacromial space will be performed using greyscale US (transverse/ longitudinal). Thickness measures and subacromial space parameters are potentially important factors in understanding the pathogenesis of rotator cuff pathology [110, 111] and tendinopathy [112]. Furthermore, power Doppler US is used for measuring vascularization as a sign of pathology and healing [22, 113]. The subject is seated upright on a chair for all US measurements with respectively shoulder internal rotation, arm resting at the side, and respectively 45 or 60° of active shoulder elevation in the scapular plane with 90° flexed elbow. The US examination is performed using a GE Healthcare LOGIQ E10 scanner (Milwaukee, Wisconsin, U.S.A.) and a ML6-15-D Matrix Linear Array Probe which is a 15-MHz frequency 50-mm transducer.

Demographic data includes age, gender, pre-injury sports/ recreational activity level, habitual and current work ability. Selected demographics and baseline measures will be used as covariates in the statistical analysis.

Outcomes in the qualitative sub-study will be interviews which focus on themes such as motivating factors and barriers to training, empowerment, and explanatory factors [46, 47]. During the process, the material can be supplemented if important concepts and themes were not included in the developed interview guide and if new facets of the research question arise.

### Participant timeline

Participants will undergo a structured time schedule including the intervention and assessments (Fig. 2).

### Sample size

The sample size is calculated to test the superiority of PAllow over PAvoid based on the change in the SPADI from baseline to week 26 (primary outcome) [79]. A total of 35 patients are required per group to establish a clinically significant mean difference of 10 points with a common standard deviation of 15 (0–100 scale) [25, 42]. We aim at having a power of 80% to verify an effect equal to or higher than the MCID of 10 points on SPADI [85, 86] at a 5% significance level. To account for dropouts (max 20%), we are planning to include a total of 42 per group.

### Recruitment

Participants are recruited from January 2022 to approximately January 2024. Recruitment is not interrupted until the desired number is achieved. All participants have a medical referral to one of the clinics.

## Assignment of interventions: allocation

### Sequence generation

The allocation sequence is computer-generated with permuted random blocks, set up by a data manager outside the project. Participants are randomly assigned to either PAllow or PAvoid with a 1:1 allocation ratio. To counter potential imbalance in the randomization both stratification and blocking are employed. Stratification by including department (department of sports medicine/ department of occupational medicine) is necessary because of possible differences in clinical practices, and furthermore stratification by sex and age (± 35 years) are employed.

### Concealment mechanism

Randomization is performed in REDCap by a secretary with no clinical involvement in the trial. The secretary will check information on age, sex, and clinic and then adds the next randomization label from the correct table in REDCap. The intervention physiotherapist login to the two-step password-protected REDCap Database to get notification of the treatment allocation. To ensure allocation concealment, the PI is blinded to block sizes and unaware of the next assignment in the allocation sequence.

### Implementation

All eligible patients who fulfil the inclusion criteria and who give consent to participate are scheduled a time for baseline testing. After consent, the secretary completes the randomization without any influence of the PI or intervention physiotherapists. After the baseline testing session, the first training session is completed. During the project, the PI will contact the intervention physiotherapist to make sure that all practical aspects run smoothly, including appointment bookings.

## Assignment of interventions: blinding

### Who will be blinded

The PI who is also the outcome assessor is kept blinded from group allocation during the entire study, and patients are requested not to disclose their allocation when outcomes are assessed at 26 weeks and 1 year follow-up. As this is an “open-label” trial the intervention physiotherapists and the patients are not blinded to treatment allocation.

### Procedure for unblinding if needed

The PI can only be un-blinded if deemed necessary, e.g., in the case of (serious) adverse events that require this otherwise blinded person to be involved in the solution of the event.

## Data collection and management

### Plans for assessment and collection of outcomes

Before starting data collection, the outcome assessor (PI) practiced and validated all objective outcome measurements according to rigorously described study protocols. Participants will complete the self-reported measurements directly into REDCap, and outcome assessor will enter the data in REDCap from the objective measurements immediately after these have been completed.

Data collection will be performed in an undisturbed room. At supervised exercise sessions, the intervention physiotherapists register the data concerning the exercise session (load management, progressions, compliance, and pain level) in the electronic medical patient record Hyperspace® EpicCare. Throughout the intervention period (0–26 weeks), the participants register details on pain level, exercise category and number, load, repetitions, and sets in a printed pain and exercise logbook. All forms were designed by the PI.

### Plans to promote participant retention and complete follow-up

In REDCap, the dashboard is monitoring whether the participants answer the questionnaires. For non-responders, an email reminder will be sent out 5 times with a 1-day interval, and if not answered after that the PI will contact the participants by email or phone. Two weeks before the planned 26-week and 1-year follow-up testing, the PI contacts the participants to schedule an appointment. The intervention physiotherapists are collecting the pain and exercise logbooks regularly as participants complete the 26-week intervention and thus the logbooks are divided in three: weeks 0–6; weeks 7–12; weeks 13–26.

### Data management

Data management will comply with the regulations of the Danish Data Protection Agency (approval reference number: P-2021–536). REDCap is the data collecting and storage system to accomplish the legislative requirements about management and safekeeping of data. PI developed a pre-defined codebook, and data are entered directly into REDCap with validation rules to verify data entered in a record meets the specified standards. The PI has personal access to data by a two-step confidential login. De-identified pseudo anonymous data are exported directly from REDCap to a logged drive. A statistician blinded to allocation will get access to pseudo anonymous data. In the “cleaning” process of raw data in preparation for the analysis, the “cleaning” procedures will be saved as a syntax- file (statistical commands) and data will be saved in a new file to keep the raw data file available. In the process of producing new variables, distribution characteristics of the new variables will be scrutinized and compared with the source and target variable to check for correctness of calculations. A project logbook will be created to be able to keep an overview of management procedures for the data. During data cleaning, data will be checked for duplicates, and summarized data and tabulated data will be used to identify missing data, outliers, and errors. A research assistant outside the project is type in data from the pain and exercise logbooks.

### Confidentiality

Medical information about participants in the study are confidential and kept in the electronic medical patient record Hyperspace® EpicCare which complies with international recommendations for confidential data protection. Only health professionals involved (medical doctors, physiotherapists) have access to this information and disclosure to third parties other involved health professionals is prohibited. When publishing data from this study, the presentation format will not include names, photos, personal information, or other data which may disclose the identity of participants.

### Plans for collection, laboratory evaluation and storage of biological specimens for genetic or molecular analysis in this trial/future use

Not applicable, as no such samples will be collected.

## Statistical methods

### Statistical methods for primary and secondary outcomes

The primary analysis will be performed at the primary endpoint (end of the 26-week intervention period). Data will be reported using the CONSORT 2010 statement: updated guidelines for reporting parallel group randomized trials. The primary efficacy analysis performed is assessment of the between-group difference in change in the SPADI score after 26 weeks in the ITT population. ITT population is defined as all randomized participants irrespective of compliance or withdrawals. A patient will be considered randomized as soon as intervention/training group is assigned by according to the allocation sequence.

Summary tables for quantitative variables included in publication are expected to include at least mean and standard deviation (SD) by treatment group. All summary tables for qualitative variables will display counts and percentages by treatment group.

The mean outcome at each of the study time points, which includes the primary outcome SPADI at week 26, will be analyzed in linear regression models; the models will be parameterized such that they directly produce inference on the difference between the randomization groups at each of the follow-up time points, beyond the difference that may be present at baseline. The analyses will account for repeated measurement by means of Generalized Estimating Equations (GEE). Furthermore, the analyses are adjusted for the stratification variables: sex, age, and clinic. Predictors of health outcomes will be analyzed with multiple regression models as independent variables, with SPADI change as dependent variable. An alpha level of 0.05 (two-sided) will be considered as being statistically significant. The statistician is blinded to the allocated interventions for the analysis. Data analysis is performed in SAS version 9.4 and R version 4.3.1.

In the qualitative sub-study, the data from the interviews are transcribed and analyzed using the template method as a starting point for the cross-sectional comparative analysis [114]. Thereby, a priori themes from theory and empiricism are included in the development of the interview guide, which expresses an initial, but not adequate and not hierarchically ordered, template for containing the interview material. There will be an ongoing iterative analysis with researcher triangulation until data saturation is achieved. The condition of tendinopathy is anchored in an interactive model for recovery specifically aimed at RC tendinopathy developed by Vila-Dieguez 2023 and which involves four overall biopsychosocial domains: tendon structure, neuromuscular processes, pain and sensorimotor processing, and psychosocial dimensions [46]. The transfer theory is used to in the understanding of the most important factors that negatively influence or positively increase the transfer between context (the intervention context and the everyday context) [115, 116]. The theories are used as components of the Template method to partly inform the development of the interview guide and as a theoretical abstraction of the qualitative analysis and in the light of the selected disease model.

### Interim analyses

No formal stopping guidelines or interim analyses are planned.

### Methods for additional analyses (e.g., subgroup analyses)

The per-protocol (PP) population is defined as participants who adhere to this protocol, defined by the following criteria in both groups: have attended at least 75% of the scheduled on-site physiotherapist appointments; have 75% compliance to pain perception during and after exercises (documented by pain logbook); have a minimum of 6 home-based training sessions weekly for the first 16 weeks of the 26 weeks intervention (documented by the exercise logbook). Further, it requires that participants do not engage in concomitant supervised exercise-based treatment for the shoulder and do not receive new, important interventions (e.g., no surgery, no shock-wave therapy or corticosteroid injection) in the main trial phase.

### Methods in analysis to handle protocol non-adherence and any statistical methods to handle missing data

The presence of missing data at the various study time points is modelled by logistic regression models using age, sex, clinic, group allocation (masked), and baseline value as explanatory variables. Estimated probabilities for the data being not missing from these models will be used as inverse probability weights (IPW) to account for possible differential attrition; GEE adjusts inference to account for the weights [117].

### Plans to give access to the full protocol, participant-level data and statistical code

The full protocol is provided in this paper. Upon publication of the planned research papers, we intend to share the de-identified data for future research purposes upon reasonable requests.

## Oversight and monitoring

### Composition of the coordinating center and trial steering committee

BHK, AMC, and PM are members of the Steering Committee and are responsible for taking decisions about major changes needed once the study has been initiated. We have no other committees involved in the oversight of the RCT. The PI (BHK) checks that recruitment is progressing sufficiently, and on a weekly basis data quality and completeness. PI also continuously instructs intervention physiotherapists as needed.

### Composition of the data monitoring committee, its role and reporting structure

Since adverse events are expected to be minimal and the intervention is not considered a high-risk intervention, no data monitoring committee is established. The physiotherapists are asked to report any adverse event to the PI, who will report these to the ethics committee. Further, if a patient during a period of training experiences worsening of symptoms exceeding expected pain or symptoms the intervention physiotherapists are reporting it to the PI, and the patient can be offered a second evaluation by the medical doctor (FJ).

### Adverse event reporting and harms

An adverse event (AE) is defined as any untoward health-related occurrence in a study participant, which does not necessarily have a causal relationship with the allocated treatment. An AE can therefore be any unfavorable and unintended sign, symptom, or disease temporally associated with the study intervention, whether the event is considered causally related to the treatment. The PI will use the following definitions to rate the severity of each AE: A mild AE is transient and easily tolerated by the participant and cover symptom flare up and exercise induced fatigue; a moderate AE causes the participant discomfort and interrupts the participant’s usual activities; a severe AE causes considerable interference with the participant’s usual activities and may be disabling and causing permanent damage. The treating intervention physiotherapists are familiar with the modification guidelines to reduce the exercise load, if participants experience short-lasting minor AEs. Acute increase in shoulder symptoms, such as severe shoulder pain (e.g., 8 or higher on the NPRS), including pain during rest will be reported to the PI by participants and/or intervention physiotherapists. As a safety precaution, if a medical evaluation is indicated, the participants will be referred to the medical doctor (FJ). Serious AEs will be reported to the Regional Committee on Health Research Ethics for the Capital Region within 7 days after the PI or others from the Steering Committee have become aware of the incident without being unblinded. Serious AEs will be assessed by health professionals outside the project for possible connection with the assessment and/or intervention in the project, but all AEs will be reported irrespective of their relationship with assessment or intervention. In case of acute injury during the project assessment or intervention, the participants will be able to seek compensation from The Danish Patient Compensations Association and/or by making a complaint to The National Agency for Patients’ Rights and Complaints. We will report the number of patients experiencing mild, moderate, and serious AEs during the interventions. An elective surgery/procedure in another anatomical region than the shoulder scheduled to occur during the study will not be considered an AE if the surgery/procedure is being performed for a pre-existing condition. However, if the pre-existing condition deteriorates unexpectedly during the study, then the deterioration of the condition for which the elective surgery/procedure is carried out will be considered an AE.

### Frequency and plans for auditing trial conduct

The Regional Committees on Health Research Ethics are annually selecting some studies for auditing. The audit process is independent of investigators and sponsors.

### Plans for communicating important protocol amendments to relevant parties (e.g., trial participants, ethical committees)

Protocol modifications decided by the Steering Committee will be reported to the Regional Committees on Health Research Ethics for the Capital Region and changes will be added to the ClinicalTrials.gov protocol.

### Dissemination plans

All results from the study, both positive, negative, and inconclusive, will be published in relevant international scientific peer-reviewed journals, with authorship following the International Committee of Medical Journal Editors (ICMJE) guidelines for publication. Results will be presented at relevant national and international conferences and to relevant patient associations and will be communicated to participants and the public in general through the media and workshops.

## Discussion

RC tendinopathy (also labelled as subacromial pain syndrome or RC-related shoulder pain) is the most common shoulder complaint in a very broad group of patients ranging from young sports active to middle aged with high occupational shoulder demands. There is a growing body of evidence for exercise therapy as the first-line treatment to improve pain, mobility, and function. However, several recent systematic reviews have emphasized the need for ongoing research to provide guidance regarding pain in relation to volume and type of exercise also with attention to the broad patient characteristics [19, 20, 118].

Furthermore, there are still many unanswered questions indicating the need for future studies, especially focusing on pain acceptance and tolerance, mechanisms of recovery, training adherence, empowerment, and transferability to everyday life in patients with RC tendinopathy [46].

Even though exercise therapy is recommended as the primary treatment option due to, among other things, its clinical effectiveness (equivalent to surgery) and cost-effectiveness (less expensive than surgery) [20], the big shortcoming is that many RCTs and systematic reviews do not describe the exercise program in detail, and it becomes unclear what constitutes the most appropriate exercise regimen. With this protocol for the PASE trial, we therefore aim to be transparent about the content of our exercise intervention including one of the unanswered questions namely whether treatment for patients with RT tendinopathy should be designed around pain based on tendon loading.

Provoking or avoiding pain during exercise can be done by increasing the external resistance for a given exercise (same exercises in the 2 programs performed with different load), assuming that more external resistance increases possible pain development. Alternatively, it can be done by varying the tendon load based on selective muscle activation during the exercises, choosing exercises meant to load or unload the tendon (tendon load based on muscle contraction determined by EMG, assuming that higher EMG activity during an exercise increases tendon load). Finally, as a 3rd option, it can be done by choosing exercises based on provocation or reduction of the patient’s symptoms during the required movement (often based on assumed biomechanical components resulting in “good” or “bad” kinematics) [69, 70]. It is obvious that the 3rd option is not acceptable from a clinical and ethical perspective. As an example, it is known that symptom provocations test (such as the empty can test [59]) are supposed to provoke pain and therefore they will never be used for exercises. It is therefore generally accepted that the “full can” position [66] exercise is selected in rehabilitation because of the “empty can” position exercise is unfavorable for glenohumeral and scapular kinematics. The 1st option—increasing or decreasing the external load—is not preferable in a RCT study like this, since it will be impossible to answer the research question about pain itself. If pain allowance is associated with external resistance, we will never know if any differences in outcome are related to differences in load rather than differences in pain production. Therefore, as in the 2nd option, the best way to design 2 exercise programs in which pain is the intervention variable, is to consider the assumed tendon load, combined with pain experience during the exercise which is our purpose.

Tissue irritability is also a significant factor in exercise therapy, and it is especially important when exercising into pain. Although tissue irritability has been included in different clinical approaches for shoulder rehabilitation [16, 119], it has not been specifically addressed in clinical trials. It will be interesting to see if the participants in the PAllow group are compliant in terms of completing training with pain of 3–5 on the NPRS. According to a feasibility study just published, a significant proportion of patients did not adhere to a 9-week program of exercise into pain of 4–7/10 [120]. We anticipate that there may be problems in motivating the patients to exercise into pain due to the obvious discomfort it causes, and this suggests that “exercise into pain” might not be applicable to all patients.

When a patient with RC tendinopathy consults a physiotherapist, it is often necessary to address other factors that influence the outcome of treatment, and not just exercise. This may be advice on reducing occupational or sports-related shoulder loads. A deeper understanding of the spectrum of mechanisms can be used to inform treatment approaches that address these patient-specific factors in an informed and targeted management strategy [47]. This will enable clinicians to deliver the right treatment approach for the right patient and the right time [48, 49].

The actual experience of and perspectives on participating in exercise programs has only been studied very sparsely among RC tendinopathy patients, yet it should be of great value to us as clinicians [44, 51]. With the qualitative sub-study, we would like to contribute knowledge about the patients’ experience, and perspective on allowing pain versus avoiding pain based on tendon loading during an exercise regimen for RC tendinopathy.

The pragmatic approach in this study using broad eligibility criteria, a consecutive sampling strategy, active exercise-based treatment intervention as a basis for comparison, and patients recruited from clinics with full patient flow and not just restricted to study participants likely to be highly responsive to the intervention will improve the generalizability and applicability of the study results. Thus, we recruit patients from well-established specialized University Hospital-based Sports Medicine and Occupational Medicine Clinic which per see also capture a broad sample of patients with RC tendinopathy.

If the PAllow intervention is found to be effective, it will potentially expand treatment options for this patient group, as there is currently no consensus [26, 40, 42–44] and the general understanding is that pain during shoulder exercise should be minimized in this patient group. By having two active exercise-based treatments, we ensure a high ethical standard of research and avoid offering redundant treatments to the patients.

The pre-registration at ClinicalTrials.gov and publication of this study protocol, including intervention transparency and thoroughly described exercise protocols greatly improve the overall quality of the current trial, and results can be easily implemented in clinical practice and guide the development of future clinical recommendations and be potential for implementation.

## Trial status

o Protocol version 1.3_30-08–2021
o Recruitment was initiated on 11 January 2022
o Completed recruitment on 5  January 2024
o Recruitment status: active, not recruiting

## Consent for publication

Written informed consent for publication of images in the exercise manuals was obtained from the person appearing in the photos. The participant information materials and informed consent form are available from the corresponding author on request.

## Competing interests

The authors declare that they have no competing interests.

### Supplementary Information

**Supplementary file 1.**

**Supplementary file 2.**

**Supplementary file 3.**

**Supplementary file 4.**

## Data Availability

The PI and PM will have full access to the data with a personal confidential password. Copenhagen University Hospital Bispebjerg Frederiksberg owns all research data collected as part of this project. The PI takes responsibility for all data collection, management, and sharing of research data. The datasets generated during the trial will not be publicly available due to regulations set out by the Danish Data Protection Agency. It will be possible to re-use the data for further research, document the validity of the research, and make possibilities for data-sharing and collaborations upon reasonable request.

## Abbreviations

AAROM: Assisted active range of motion
ABD: Abduction
AC: Acromioclavicular
AE: Adverse event
AHD: Acromiohumeral distance
ANCOVA: Analysis of covariance
AROM: Active range of motion
BFH: Bispebjerg and Frederiksberg Hospital
CERT: Consensus on Exercise Reporting Template
CONSORT: Consolidated Standards of Reporting Trial
EMG: Electromyography
EQUATOR: Enhancing the quality and transparency of health research
ER: External rotation
EXT: Extension
FLEX: Flexion
GEE: Generalized Estimating Equations
GRS: Global Rating Scale
HRQOL: Health-related quality of life
ICMJE: International Committee of Medical Journal Editors
IPW: Inverse probability weights
IR: Internal rotation
ITT: Intention to treat
MCID: Minimal clinically important difference
MVC: Maximal voluntary contraction
NPRS: Numerical Pain Rating Scale
NV: Neovascularity
PAllow: Pain allowance program
PASE: PAin during Shoulder Exercise
PASS: Patient acceptable symptom state
PAvoid: Pain avoidance program
PI: Principal investigator
PP: Per protocol
PPT: Pressure pain threshold
PRO: Patient-reported outcomes
PROM: Passive range of motion
Quick DASH: Short form of Disabilities Arm, Shoulder and Hand questionnaire
RC: Rotator cuff
RCT: Randomized controlled trial
REDCap: Research Electronic Data Capture
RM: Repetition maximum
ROM: Range of motion
SAS: Subacromial space
SD: Standard deviation
SPADI: Shoulder Pain And Disability Index
SPIRIT: Standard Protocol Items: Recommendation For Interventional Trials
SST: Supraspinatus tendon
TIDieR: Template for Intervention Description and Replication
US: Ultrasound
WAI: Work Ability Index
MVC: Maximal voluntary contraction
NPRS: Numeric Pain Rating Scale
