# Supplementary material for: To allow or avoid pain during shoulder rehabilitation exercises for patients with chronic rotator cuff tendinopathy-Study protocol for a randomized controlled trial (the PASE trial)
Source: Trials. 2024 Feb 21;25:135. doi: 10.1186/s13063-024-07973-6 (PMC10880378; doi:10.1186/s13063-024-07973-6)
Supplement: Supplementary file 2 [file 13063_2024_7973_MOESM2_ESM.pdf]

CERT ✓ Consensus on Exercise Reporting Template

A Checklist for what to include when reporting exercise programs

Section/Topic	Item #	Checklist item	Location **	
			Primary paper (page, table, appendix)	† Other (paper or protocol, website (URL)
**WHAT: materials**	1	Detailed description of the type of exercise equipment (e.g. weights, exercise equipment such as machines, treadmill, bicycle ergometer etc)	16	
**WHO: provider**	2	Detailed description of the qualifications, teaching/supervising expertise, and/or training undertaken by the exercise instructor	16	
**HOW: delivery**	3	Describe whether exercises are performed individually or in a group	15, table 1	
	4	Describe whether exercises are supervised or unsupervised and how they are delivered	15, table 1	
	5	Detailed description of how adherence to exercise is measured and reported	16, 17	
	6	Detailed description of motivation strategies	17	
	7a	Detailed description of the decision rule(s) for determining exercise progression	15	
	7b	Detailed description of how the exercise program was progressed	15, suppl 3 & 4	
	8	Detailed description of each exercise to enable replication (e.g. photographs, illustrations , video etc)	suppl 3 & 4	
	9	Detailed description of any home program component (e.g. other exercises, stretching etc)	suppl 3 & 4	
	10	Describe whether there are any non-exercise components (e.g. education, cognitive behavioural therapy, massage etc)	13	
	11	Describe the type and number of adverse events that occurred during exercise	N/A	
**WHERE: location**	12	Describe the setting in which the exercises are performed	15	
**WHEN, HOW MUCH: dosage**	13	Detailed description of the exercise intervention including, but not limited to, number of exercise repetitions/sets/sessions, session duration, intervention/program duration etc	15, 16, figure 3	
**TAILORING: what, how**	14a	Describe whether the exercises are generic (one size fits all) or tailored whether tailored to the individual	13, table 1	
	14b	Detailed description of how exercises are tailored to the individual	13	
	15	Describe the decision rule for determining the starting level at which people commence an exercise program (such as beginner, intermediate, advanced etc)	13	
**HOW WELL: planned, actual**	16a	Describe how adherence or fidelity to the exercise intervention is assessed/measured	17	
	16b	Describe the extent to which the intervention was delivered as planned	N/A	

***It is recommended that this checklist is used in conjunction with the Explanation and Elaboration Statement which is a guide each item in the CERT Checklist**

The CERT Checklist is designed for reporting details of an exercise intervention. The CERT Checklist should be used in conjunction with a reporting checklist appropriate for the study type e.g. the CONSORT Statement (www.consort-statement.org) for randomised controlled trials, the SPIRIT Statement (www.spirit-statement.org) for a clinical trial protocol. For further guidance regarding reporting guidelines please consult the EQUATOR network (www.equator-network.org)

** Authors – please use N/A if an item is not applicable Reviewers – please use "?" if information is not provided or not/insufficiently reported

† If the information is not provided in the primary paper that is under consideration, please provide details of where this information is available e.g. in a published protocol, published papers (provide citation details) or on a website (provide the URL).
