# Supplementary material for: To allow or avoid pain during shoulder rehabilitation exercises for patients with chronic rotator cuff tendinopathy-Study protocol for a randomized controlled trial (the PASE trial)
Source: Trials. 2024 Feb 21;25:135. doi: 10.1186/s13063-024-07973-6 (PMC10880378; doi:10.1186/s13063-024-07973-6)
Supplement: Supplementary file 3 [file 13063_2024_7973_MOESM3_ESM.pdf]

Kjær et al.
Supplementary file 3

Exercise manual and pain management guidelines for the PAin during Shoulder Exercise trial (the PASE trial)

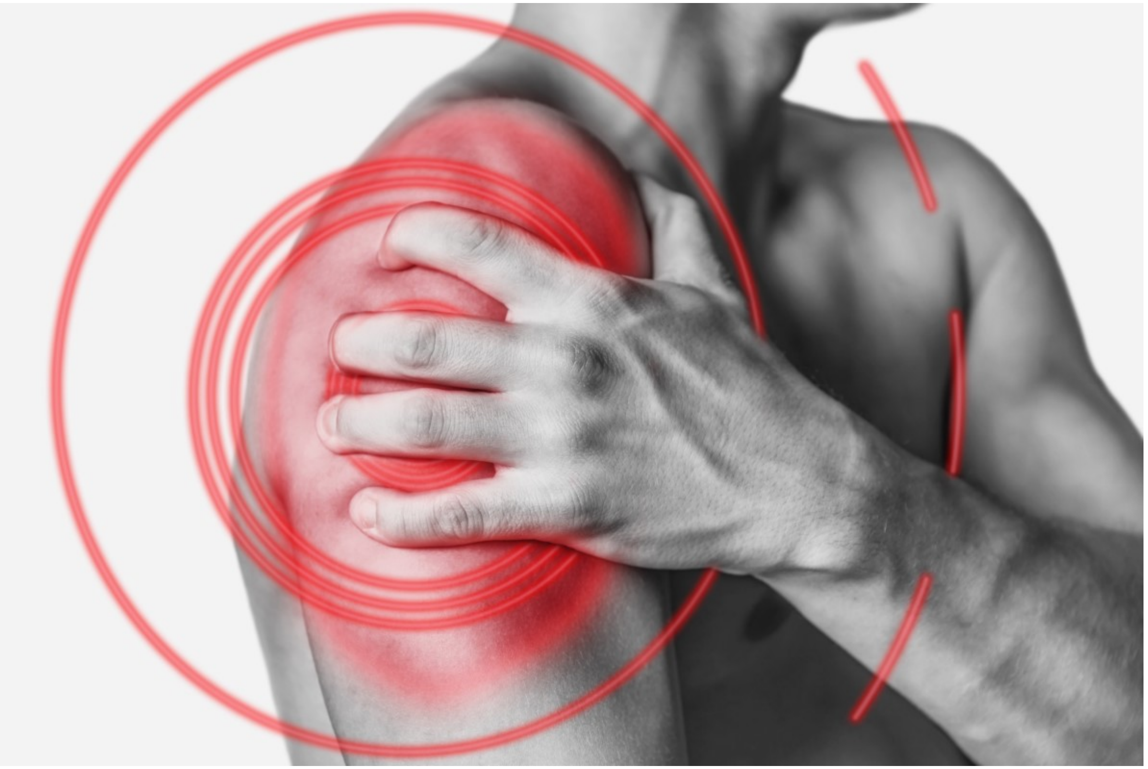

Principal investigator: Birgitte Hougs Kjær Ph.D, Physiotherapist
Department of Physical and Occupational Therapy, Copenhagen University Hospital Bispebjerg and Frederiksberg
Phone +45 3863 7200, Birgitte.Hougs.Kjaer@regionh.dk

GENERAL INFORMATION ABOUT THE EXERCISE PROGRAMME

1. Management of shoulder symptoms during home exercises
2. Pain monitoring
3. Progression and exercises
4. Other progression parameters

1. Management of shoulder symptoms during exercises

Management of shoulder pain and/or symptoms during exercises is performed with the use of a symptom-scale depicted below. The scale ranges from 0-10, with 10 being worst imaginable symptoms.

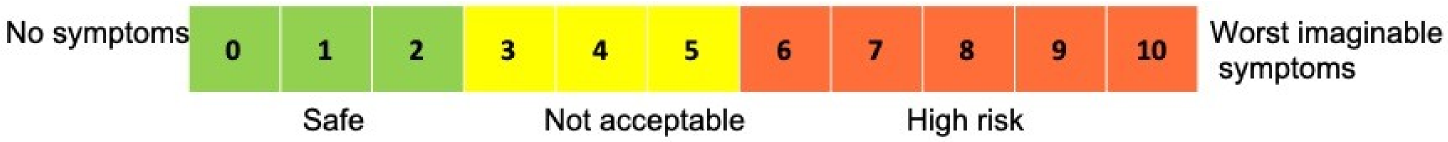

The cutoff regarding progression in relation to pain perception during and after exercises is ≤2/10.

2. Pain monitoring

Self-reported pain levels will be registered in a pain diary.
In connection with a training session, you are asked to report pain level

- before
- during
- immediately after
- 1 hour after
- the morning after

3. Progression and exercises

Exercise A-E are performed for the first 6 weeks. From week 7 exercise F-K are performed.

Repetitions: 3 times 15 repetitions with pain as explained above.
How many times a day/week? In general exercises should be performed daily.

- Week 0: Two exercises (1 exercise in the morning and 1 in the evening)
- Week 1: Add 1 exercise = 3 exercises
- Week 4 and on: 4 exercises (varying the exercises from week to week)

4. Other progression parameters

To allow for progression each exercise has between 3 and 18 levels of difficulty.
For all exercises several progression types are possible:

- increasing resistance
- changing the plane
- ball versus towel
- increasing ROM
- increasing kinetic chain demands (perform exercise standing on one leg, or squatting)

An exercise may be progressed when satisfactory neuromuscular control is obtained, according to the following criteria:

- it can be easily performed with good movement quality
- resistance and number of repetitions are achieved
- there are no additional accompanying movements in the body
- it can be performed within the permitted pain limit
- it can be performed with steady breathing and general body control
- It can be performed without need for visual, verbal, or tactile feedback

PAvoid A Sliding exercise

Biomechanical purpose: To perform elevation of arm/ forward flexion in close kinetic chain.

Generally: For all bench slides the plane may be changed sagittal versus scapular to avoid pain.
Avoid compensating strategies such as shrugging/excessive shoulder elevation, too much flexion in the trunk, head position too much into flexion (protracted) during the exercises.
For all stages with resistance, another progression is possible by increasing strength in elastic band.

Purpose from a patient perspective: The exercises in this category are all loading the shoulder tendons minimally although lots of movement and resistance. They are important for a good shoulder function, since the shoulder blades and shoulder joints are a constant interplay.

Level	Exercise	Material			Description
1	Bilateral bench slide ball <90°	Ball	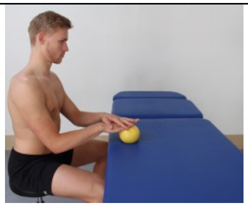	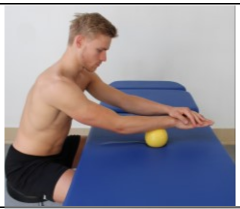	Sitting erect on a chair in front of table/ bench. The subject rolls the ball with both hands forward to <90°of glenohumeral (GH) forward flexion (FF) and back to the starting position.
2	Bilateral bench slide towel <90°	Towel	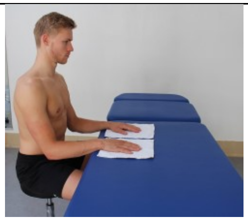	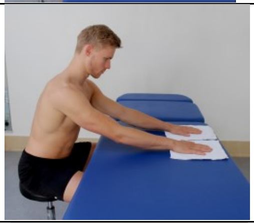	Sitting erect on a chair in front of table/ bench. The subject slides the towel with both hands forward to <90° of GH FF and back to the starting position.
3	Bilateral bench slide with resistance around back <90°	Elastic band (1.5m)	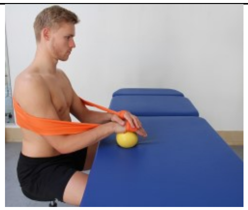	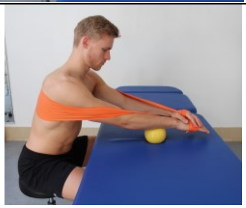	Sitting erect on a chair in front of table/ bench. The subject *place a long elastic band around back and holding onto it while rolling* the ball with both hands forward to <90° of GH FF and back to the starting position. Make 2 loops at both ends of the elastic band to put 4 fingers in it (so the fingers can stay extended while they glide over the ball)

4	Bilateral bench slide ball >90°	Ball	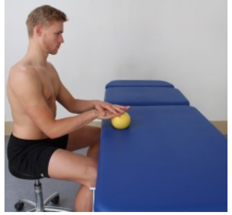	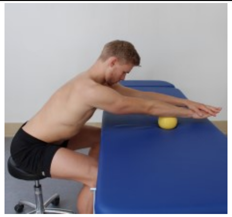	Sitting erect on a chair in front of table/ bench. The subject rolls the ball with both hands forward to >90° of glenohumeral (GH) forward flexion (FF) and back to the starting position.
5	Bilateral bench slide towel >90°	Towel	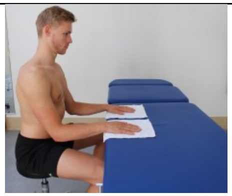	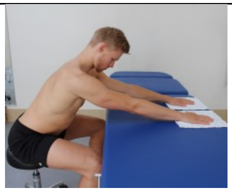	Sitting erect on a chair in front of table/ bench. The subject slides the towel with both hands forward to 120° of GH FF and back to the starting position.
6	Bilateral bench slide with resistance around back >90°	Elastic band (1.5m)	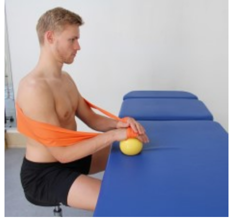	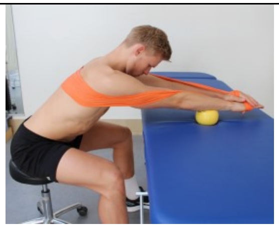	Sitting erect on a chair in front of table/ bench. The subject place a long elastic band around back below level of shoulders and holding onto it while rolling the ball with both hands forward to 120° of glenohumeral (GH) forward flexion (FF) and back to the starting position.
7	Unilateral bench slide < 90° with ball	Ball	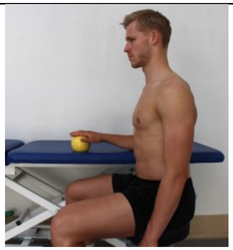	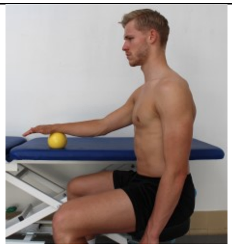	Sitting erect on a chair next to a table/ bench. With the hand of the affected shoulder the subject rolls the ball forward to 60° of GH FF and back to the starting position.
8	Unilateral bench slide < 90° with towel	Towel	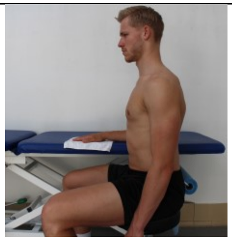	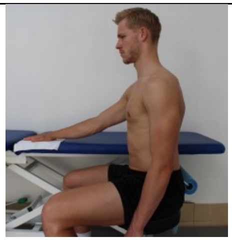	Sitting erect on a chair next to a table/ bench. With the hand of the affected shoulder the subject slides the towel forward to 60° of GH FF and back to the starting position.

9	Unilateral bench slide < 90° with resistance with ball	Ball & Elastic band	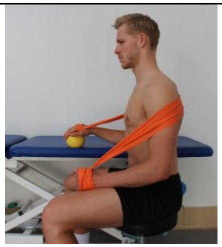	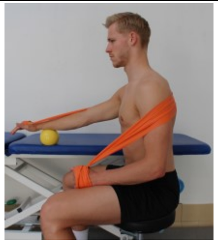	Sitting erect on a chair next to a table/ bench. With the hand of the affected shoulder the subject rolls the ball forward to 60° of GH FF with resistance from an elastic band and back to the starting position.
10	Unilateral bench slide < 90° with resistance with towel	Towel & Elastic band	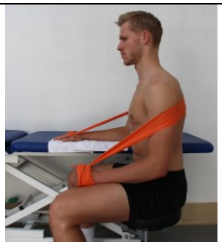	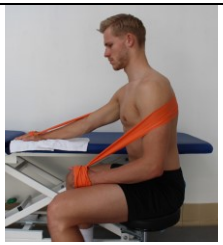	Sitting erect on a chair next to a table/ bench. With the hand of the affected shoulder the subject slides the towel forward to 60° of GH FF with resistance from an elastic band and back to the starting position.
11	Unilateral bench slide > 90° with ball	Ball	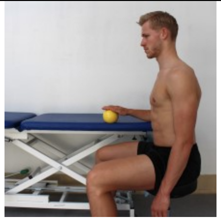	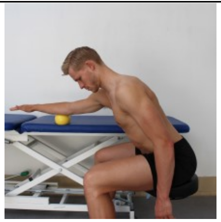	Sitting erect on a chair next to a table/ bench. With the hand of the affected shoulder the subject rolls the ball forward to 120° of GH FF and back to the starting position.<br>Can be increased to full ROM.
12	Unilateral bench slide > 90° with towel	Towel	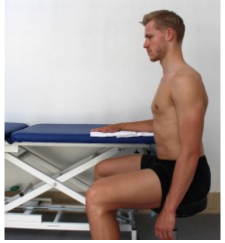	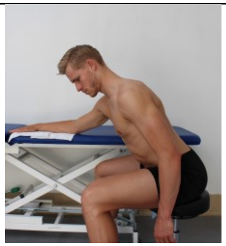	Sitting erect on a chair next to a table/ bench. With the hand of the affected shoulder the subject slides the towel forward to 120° of GH FF and back to the starting position.<br>Can be increased to full ROM.
13	Unilateral bench slide > 90° with resistance with ball	Ball & Elastic band (1.5m)	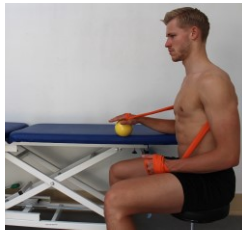	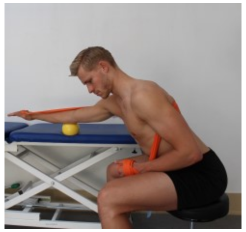	Sitting erect on a chair next to a table/ bench. With the hand of the affected shoulder the subject rolls the ball forward to 120° of GH FF with resistance from an elastic band and back to the starting position.<br>Can be increased to full ROM.<br>Further progression is possible by increasing strength in elastic band.

14	Unilateral bench slide > 90° with resistance with towel	Towel & Elastic band (1.5m)	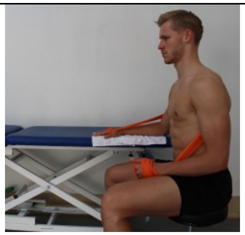	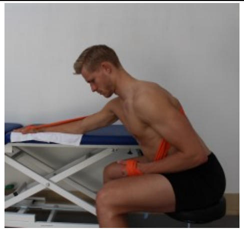	Sitting erect on a chair next to a table/ bench.<br>With the hand of the affected shoulder the subject slides the towel forward to 120° of GH FF with resistance from an elastic band and back to the starting position.<br>Can be increased to full ROM.<br>Further progression is possible by increasing strength in elastic band.
15	Wall slides with ball	Ball	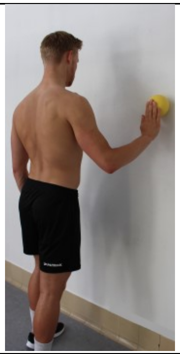	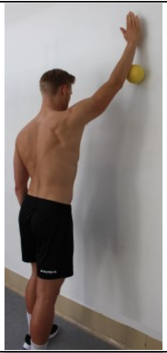	Standing facing the wall. Put your opposite foot (of the arm you want to exercise) a bit in front of the other. With the hand of the affected shoulder the subject rolls the ball on the wall in the scapular plane to 150° (or as high as possible without pain) of GH FF and back to the starting position.
16	Wall slides with towel	Towel	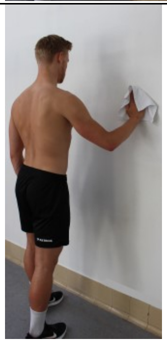	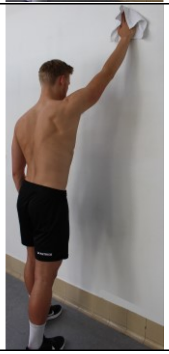	Standing facing the wall.<br>With the hand of the affected shoulder the subject<br>slides the towel on the wall in the scapular plane to 150° of GH FF and back to the starting position.

17	Wall slides with resistance with ball	Ball & Elastic band	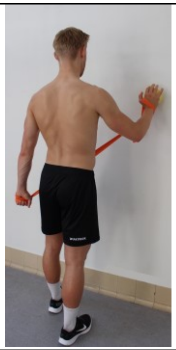	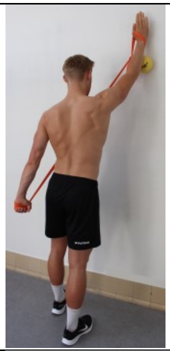	Standing facing the wall.<br>With the hand of the affected shoulder the subject rolls the ball on the wall in the scapular plane to 150° of GH FF with resistance from an elastic band and back to the starting position.<br>Further progression is possible by increasing strength in elastic band.
18	Wall slides with resistance with towel	Towel & Elastic band	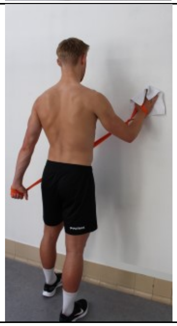	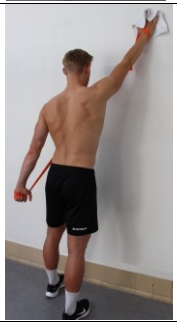	Standing facing the wall.<br>With the hand of the affected shoulder the subject slides the towel on the wall in the scapular plane to 150° of GH FF with resistance from an elastic band and back to the starting position.<br>Further progression is possible by increasing strength in elastic band.

PAvoid B Levy exercises

Biomechanical purpose: To perform exercises minimally affected by gravity with shoulder elevation of 90° +/- in open kinetic chain.

Generally: Make sure to always perform the start of the Levy exercise (the upward movement) and the end of the exercise (the downward movement) in a passive mode (with both hands in which the affected arm is supported by the unaffected) and always with a short level arm (elbow bent) *

For all stages with resistance, progression is possible by increasing the weight of the exercise ball.

Purpose from a patient perspective: The exercises in this category are meant to increase your elevation capacity, without too much loading of your tendons. After progressing through all levels, you will be able to elevate your arm above shoulder height with bended elbow, without pain.

Level	Exercise	Material	Photos	Description
1	Levy passive elevation		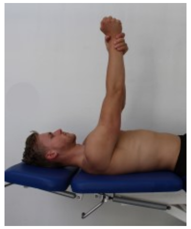 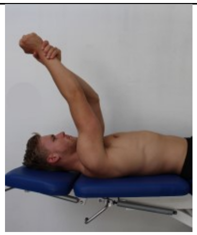 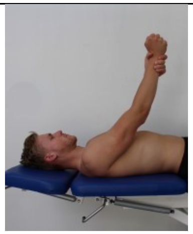	Supine lying.<br>The subject brings the arm passively* to 90° of FF and moves the arm *passively* upwards to 120° GH FF and downwards to 60° GH FF and back to the starting position.<br><br>*With help of the non-affected side
2	Levy active assisted elevation		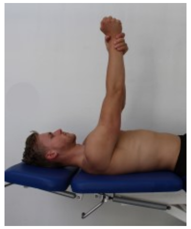 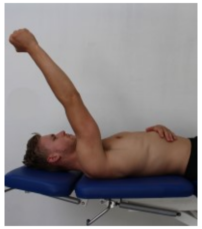 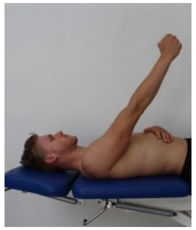	Supine lying.<br>The subject brings the arm passively* to 90° of FF and moves the arm *actively* upwards to e.g., 120° GH FF and downwards to e.g., 60° GH FF and back to the starting position. The range depends on the possible range without pain.
3	Levy active assisted elevation<br>+ resistance	Exercise weight ball	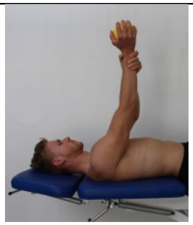 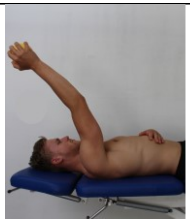 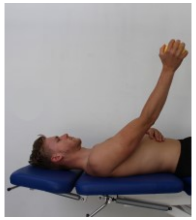	Supine lying.<br>The subject brings the arm passively* holding an exercise weight ball to 90° of FF and moves the arm actively upwards to 120° GH FF and downwards to 60° GH FF and back to the starting position.<br>Further progression is possible by increasing weight of exercise ball.
4	Levy inclined assisted elevation<br><br>*Inclination: 30°, 45°; 60°, lying supine in sofa with some pillows under upper body.*	Exercise weight ball	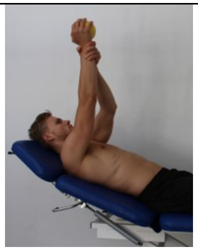 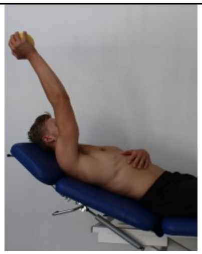 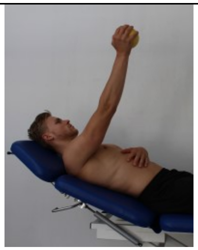	Supine inclined lying.<br>The subject brings the arm passively* to the vertical position and moves the arm actively upwards to 120° GH FF and forward to 90° GH FF and back to the starting position.

5	Levy 45° inclined assisted elevation<br>+ "unstable" resistance<br><br>*"unstable" resistance: kettle bell, half full water bottle with water held upside down*	Water bottle, or kettle bell	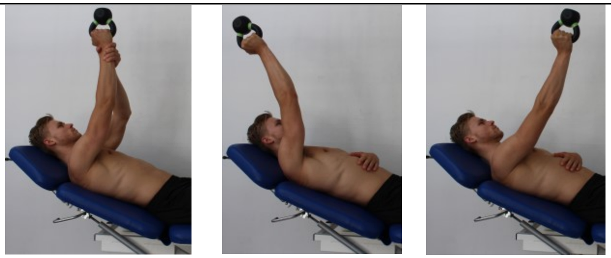	Supine inclined lying.<br>The subject brings the arm passively* holding a water bottle or kettle bell to the vertical position and moves the arm actively upwards to 120° GH FF and forward to 90° GH FF and back to the starting position.<br>Further progression is possible by changing the “unstable” resistance.
6	Levy >45° inclined assisted elevation<br>+ resistance	Exercise weight ball	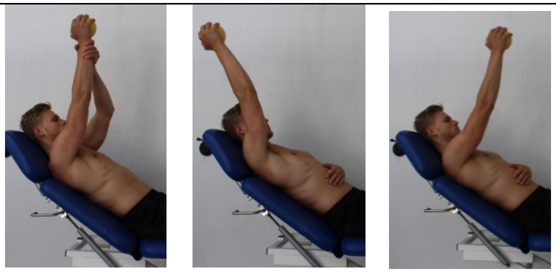	Sitting inclined.<br>The subject brings the arm passively* holding an exercise weight ball to the vertical position and moves the arm actively upwards to 120° GH FF and forward to 90° GH FF and back to the starting position.<br>Further progression is possible by increasing weight of exercise ball.
7	Levy >45° inclined active elevation with short lever arm<br>+ resistance	Exercise weight ball	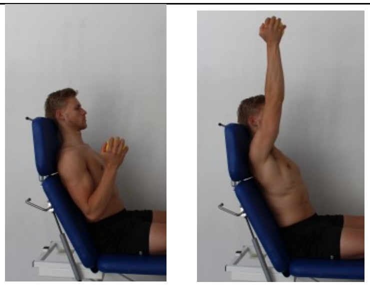	Sitting inclined.<br>Elbow bent; hand close to shoulder. Actively elevate the arm vertically as high as possible.<br>Further progression is possible by holding an exercise weight ball.<br><br>This is the first time the passive part of the exercise disappears.
8	Levy upright sitting active elevation + resistance	Exercise weight ball	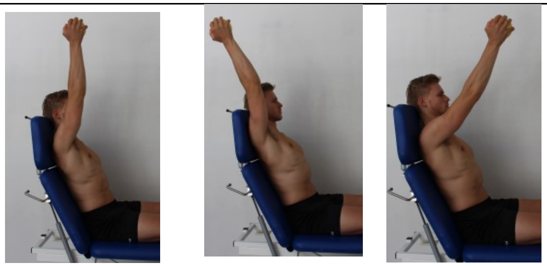	Upright sitting.<br>The subject brings the arm actively holding an exercise weight ball to the vertical position and moves the arm actively backwards to maximal GH FF and forwards to 135° GH flexion and back to the starting position.

PAvoid C Supine band

Biomechanical purpose: To perform exercises from shoulder elevation of 90° to max in open kinetic chain.

Generally:
Avoid compensating strategies such as shrugging/excessive shoulder elevation, too much flexion in the trunk, head position too much into flexion (protracted) during the exercises.
For all stages with resistance, another progression is possible by increasing strength in elastic band.

Purpose from a patient perspective: The exercises in this category are meant to increase your elevation capacity, without too much loading of your tendons. After progressing through all levels, you will be able to elevate your arm above shoulder height with bended elbow, without pain.

Level	Exercise	Material	Photos	Description
1	Supine passive elevation from 90° to max		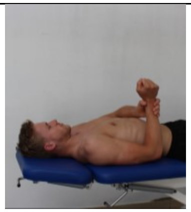 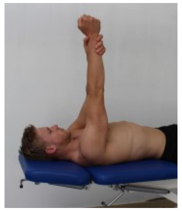 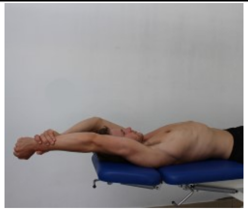	Supine lying.<br>The subject brings the arm passively* to the vertical position and moves the arm passively upwards to max GH FF and back to the starting position.<br><br>*With help of the non-affected side
2	Supine band with resistance that **assists** movement	Elastic band<br>Women<br>→ blue<br>Men<br>→ orange	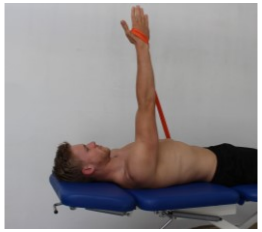 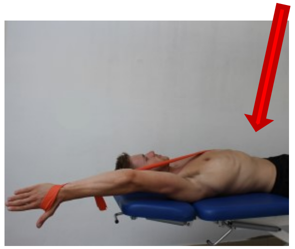	Supine lying. Opposite hand at hip holding elastic band (red arrow)<br>The subject brings the arm actively to the vertical position and then moves the arm actively upwards to max GH FF with assistance from a light elastic band and back to the starting position (the arm “falls” into FF).
3	Supine band with resistance that **resists** movement	Elastic band<br>Women<br>→ orange<br>Men<br>→ white	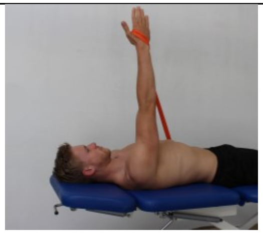 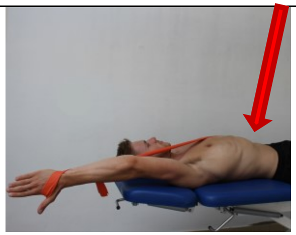	Supine lying. Opposite hand at hip holding elastic band (red arrow)<br>The subject moves the arm actively upwards to max GH FF with resistance from an elastic band and back to the starting position.<br>Further progression is possible by increasing strength in elastic band.

PAvoid D Extension/ rowing

Biomechanical purpose: To perform extension of the shoulder/ arm.

Generally:
Avoid compensating strategies such as shrugging/excessive shoulder elevation, too much flexion in the trunk, head position too much into flexion (protracted) during the exercises. Also avoid moving the shoulders forward (protraction).
For all stages with resistance from elastic band, progression is possible by increasing strength in elastic band.

Purpose from a patient perspective: The exercises in this category are all loading the shoulder tendons minimally although lots of movement and resistance. They are important for a good shoulder function, since the shoulder blades and shoulder joints are a constant interplay.

Level	Exercise	Material	Photos		Description
1	Bilateral rowing + resistance small range (20-0°) elbow bent	Elastic band	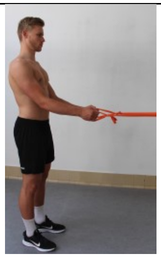	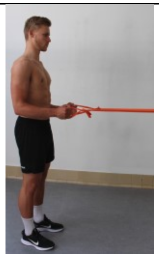	Standing.<br>Elastic band is fixed in doorhandle or rib at the level of the hip.<br>The subject moves the arm (elbow bent) actively from 20° flexion to 0° (neutral) with resistance from an elastic band and back to the starting position.
2	Bilateral rowing + resistance large range (90-0°) elbow bent	Elastic band	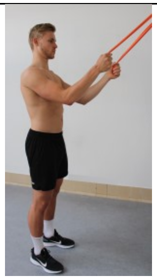	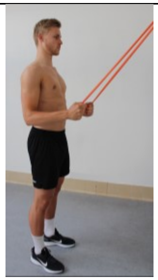	Standing.<br>Elastic band is fixed at the level of the face / head.<br>The subject moves the arm (elbow bent) actively from 90° flexion to 0° (neutral) with resistance from an elastic band and back to the starting position.
3	Bilateral rowing + resistance small range (20-0°) elbow extended	Elastic band	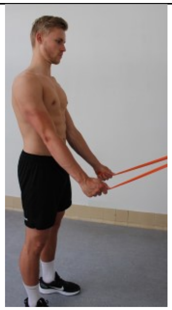	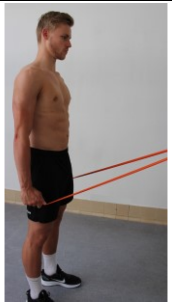	Standing.<br>Elastic band is fixed in doorhandle or rib at the level of the hip.<br>The subject moves the arm (elbow extended) actively from 20° flexion to 0° (neutral) with resistance from an elastic band and back to the starting position.

4	Bilateral rowing + resistance large range (90-0°) elbow extended	Elastic band	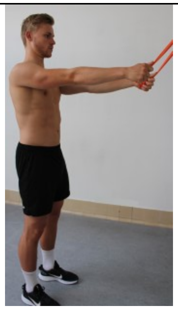	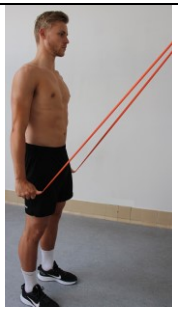	Standing.<br>Elastic band is fixed in doorhandle or rib at the level of the hip.<br>The subject moves the arm (elbow extended) actively from 90° flexion to 0° (neutral) with resistance from an elastic band and back to the starting position.
5	Prone extension without resistance		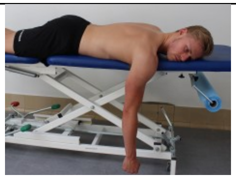	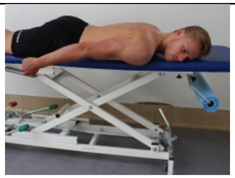	Prone lying.<br>The subject moves the arm (elbow extended) actively from 90° flexion (vertical) to 0° (horizontal) and back to the starting position.
6	Prone extension with dumbbell resistance	Dumbbell<br>Women<br>→ 2 kg<br>Men<br>→ 4 kg	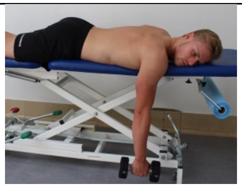	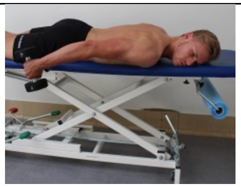	Prone lying.<br>The subject moves the arm (elbow extended) actively from 90° flexion (vertical) to 0° (horizontal) with dumbbell resistance and back to the starting position.<br>Further progression is possible by increasing weight of dumbbell.<br>The weight may decrease depending on the amount of pain (Pavoid group)
7	Prone with dumbbell resistance + ER	Dumbbell<br>Women<br>→ 2 kg<br>Men<br>→ 4 kg	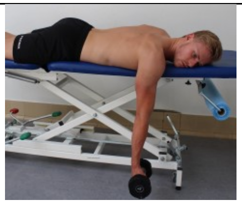	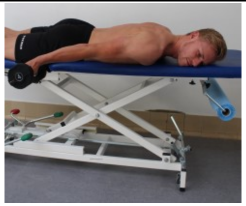	Prone lying.<br>The subject moves the arm (elbow extended) actively from 90° flexion (vertical) to 0° (horizontal) with dumbbell resistance and back to the starting position.<br>The thumb is pointing away from body (maximal external rotation).<br>Further progression is possible by increasing weight of dumbbell.

PAvoid E Exercises for level -1

Biomechanical purpose: To perform very gentle and careful exercises in close and open kinetic chain.

Generally: Avoid compensating strategies such as shrugging/excessive shoulder elevation, too much flexion in the trunk, head position too much into flexion (protracted) during the exercises. Also avoid moving the shoulders forward (protraction).

Purpose from a patient perspective: The exercises in this category are all loading the shoulder tendons minimally.

Level	Exercise	Material	Photos	Description
1	Closed chain pendulum	Ball	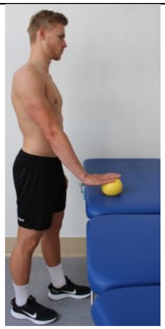 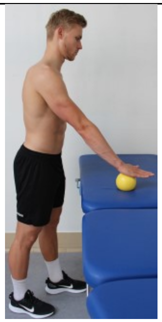	Standing in forward flexion. The elbow is stretched. The subject is rolling a ball.
2	Hand to foot		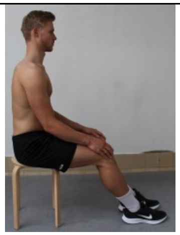 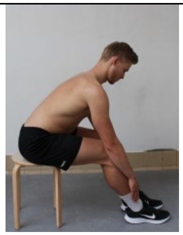 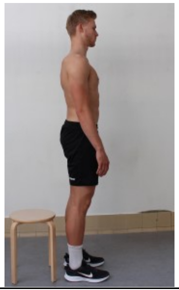 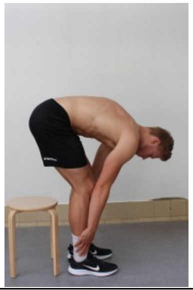	Sitting or standing.<br>The subject moves the arm toward the foot and back to the starting position.

3	Forward flexion (push hand into ball with fist)	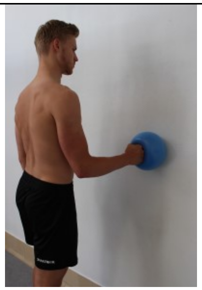	Standing facing the wall with elbow in 90° flexion. The subject performs shoulder flexion and push hand into ball against the wall.
4	Extension (push elbow into ball)	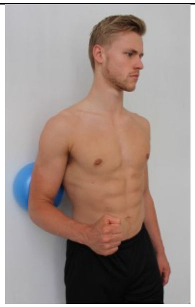	Standing with back to the wall.<br>The subject performs shoulder extension and push elbow into a ball/ squeeze a ball into the wall.

Allow F External rotation

Biomechanical purpose: To perform external rotation of the shoulder in open kinetic chain.

Generally: Avoid compensating strategies such as shrugging/excessive shoulder elevation, too much flexion in the trunk, head position too much into flexion (protracted) during the exercises. Also avoid moving the shoulders forward (protraction).
Progression is possible by adding an elastic band.

Purpose from a patient perspective: The exercises in this category are all loading the shoulder tendons. They are important for a good shoulder function, since the shoulder blades and shoulder joints are a constant interplay.

Level	Exercise	Material			Description
1	Side lying external rotation in neutral	Dumbbell	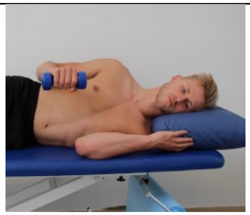	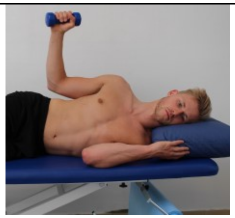	Side lying with rolled up towel or puddle pillow between elbow and trunk.<br>The subject moves the arm (elbow flexed 90°) actively from horizontal to max external rotation and back to the starting position.<br>Progression is possible by adding dumbbell.
2	Standing external rotation in neutral	Elastic band	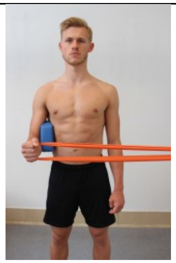	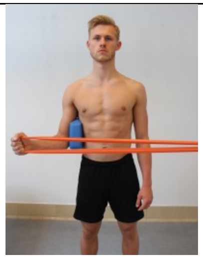	Standing with rolled-up towel or puddle pillow between elbow and trunk. The elastic band is attached to a door handle or similar.<br>Holding the elastic band, the subject moves the arm (elbow flexed 90°) actively from pointing forward to pointing in 45°-60° external rotation and back to the starting position.
3	Sitting external rotation unilateral	Elastic band	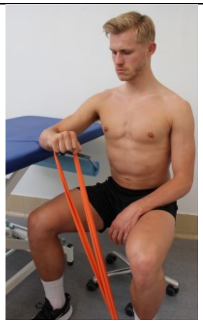	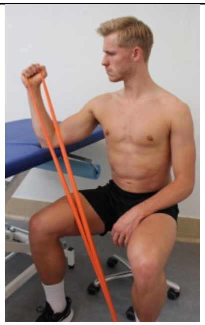	Sitting erect on a chair next to a table/ bench. The elastic band is attached to opposite foot.<br>The elbow is flexed 90° supported by table/ bench and the shoulder is in slight elevation in the scapular plane.<br>Holding the elastic band, the subject moves the arm actively from 45° internal rotation upward to max external rotation and back to the starting position.<br>Further progression is possible by increasing strength of elastic band.

4	Sitting external rotation in loose packed position bilateral (office worker exercise)	Elastic band	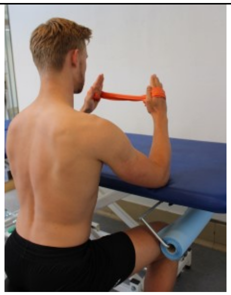	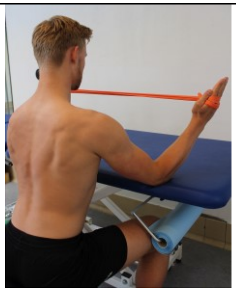	Sitting leaned forward on a chair in front of a table/ bench. The elbow is flexed 90° supported by table/ bench and the shoulder is flexed 30-45°.<br>Holding the elastic band in both hands, the subject moves the arm actively from 30° internal rotation upward to max external rotation and back to the starting position.<br>Further progression is possible by increasing strength of elastic band.
5	Sitting external rotation in 90° abduction	Elastic band	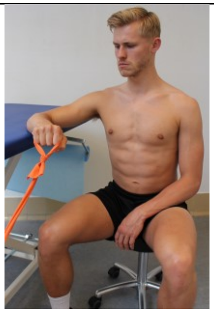	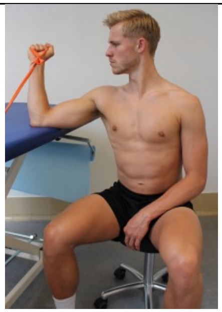	Sitting erect on a chair next to a table/ bench in shoulder level. The arm is resting on the bench with the elbow flexed 90° and shoulder flexed 90°. The elastic band is attached in front of the bench. Holding the elastic band, the subject moves the arm actively from horizontal upward to max external rotation and back to the starting position.<br>Further progression is possible by increasing strength of elastic band.
6	Prone external rotation in 90° abduction	Dumbbell	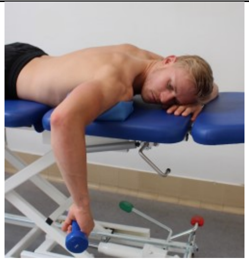	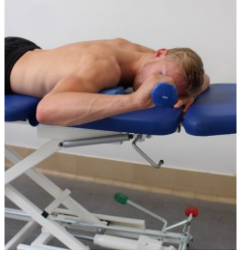	Prone lying on bench with the shoulder/ upper arm resting on a rolled-up towel or puddle pillow with elbow flexed 90° and shoulder flexed 90°.<br>The subject moves the arm actively from vertical upward to max external rotation and back to the starting position.<br>Further progression is possible by adding elastic band.

PAllow G Elevation exercises

Biomechanical purpose: To perform shoulder elevation in open kinetic chain.

Generally:
Avoid compensating strategies such as shrugging/excessive shoulder elevation, too much flexion in the trunk, head position too much into flexion (protracted) during the exercises. Also avoid moving the shoulders forward (protraction).
Progression is possible by increasing weight of dumbbell or exercise weight ball.

Purpose from a patient perspective: The exercises in this category are all loading the shoulder tendons. They are important for a good shoulder function, since the shoulder blades and shoulder joints are a constant interplay.

Level	Exercise	Material	Photos	Description
1	Full can < 90°	Dumbbell	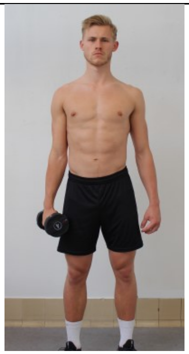 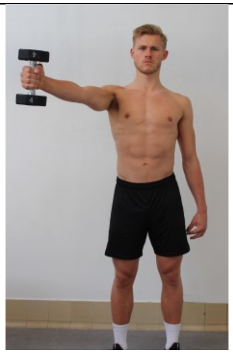	Standing. The subject performs elevation in the scapular plane in open chain to 90° of GH FF and back to the starting position.
2	Full can > 90°	Dumbbell	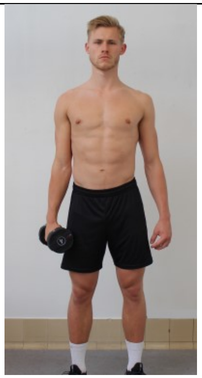 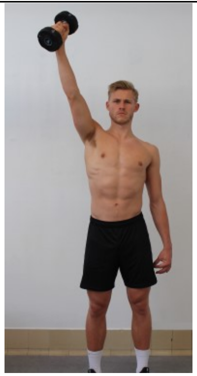	Standing. The subject performs elevation in the scapular plane in open chain to 150° of GH FF and back to the starting position.

3	Prone horizontal abd + ER	Dumbbell	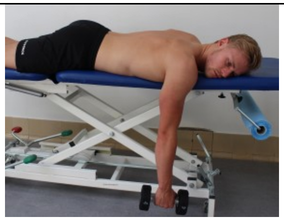 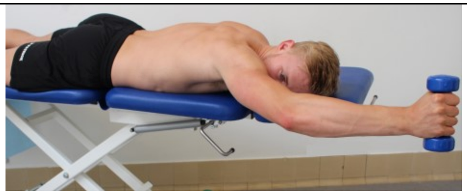	Prone lying. The subject performs a horizontal abduction + ER in open chain to 150° of GH FF and back to the starting position.
4	Prone elevation	Dumbbell	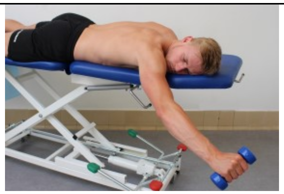 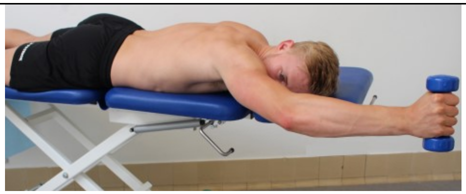	Prone lying. The subject performs elevation in the scapular plane in open chain to 150° of GH FF and back to the starting position.

PAllow H Plyometric exercises

Biomechanical purpose: To perform plyometric exercises by catching an exercise weight ball.

Generally: To add some speed to exercise program.
Progression is possible by increasing weight of exercise weight ball.

Purpose from a patient perspective: To be able to return to sport, work or e.g., gardening it is imperative to add more speed in exercises. The shoulder exercises in this category are all loading the shoulder tendons, however with more speed.

Level	Exercise	Material	Photos	Description
1	Side lying catching in neutral	Exercise weight ball	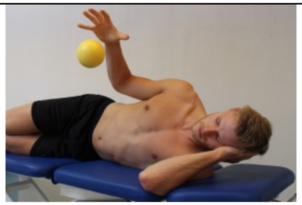 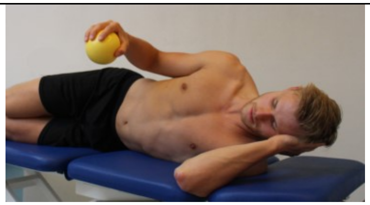	The arm along the side with elbow flexed 90° catching an exercise weight ball.

2	Side lying catching in 90° forward flexion	Exercise weight ball	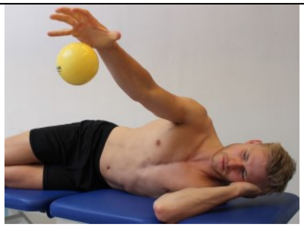	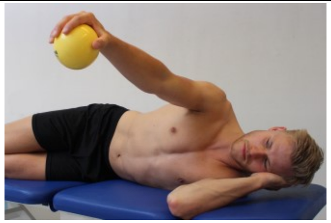	Side lying with shoulder flexed 90° and elbow stretched catching an exercise weight ball.
3	Prone catching 90°-90°	Exercise weight ball	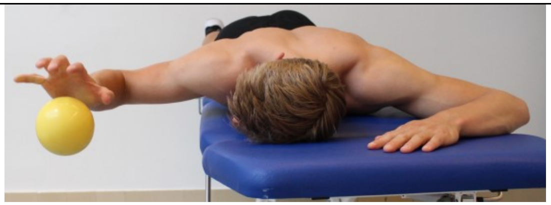<br>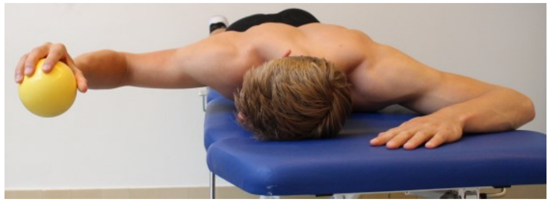		Prone lying with shoulder abducted 90°, ER to horizontal and elbow flexed 90°.<br>Subject is catching an exercise weight ball.
4	Prone catching full elevation	Exercise weight ball	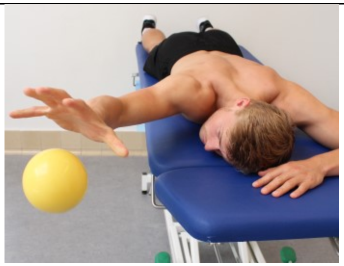	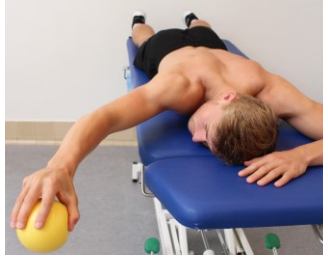	Prone lying with shoulder in full abduction.<br>Subject is catching an exercise weight ball.
5	Standing catching in 90° abduction	Exercise weight ball	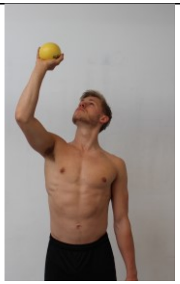		Standing with shoulder abducted 90°, ER to vertical and elbow flexed 90°.<br>Subject is catching an exercise weight ball.

PAllow IJ Eccentric exercises

Biomechanical purpose: To perform eccentric exercises, which means the tendon is loaded with a heavier weight than assumed possible in a concentric way.

Generally: Especially the eccentric part should be performed with good movement quality. Avoid compensating strategies such as shrugging/excessive shoulder elevation, too much flexion in the trunk, head position too much into flexion (protracted) and avoid moving the shoulders forward (protraction) during the exercises.
Progression is possible by increasing weight of dumbbell or exercise weight ball.

Purpose from a patient perspective: In daily life, most of the movement involve tendon loading while the muscle is lengthening (eccentric work). From a functional perspective eccentric work are relevant.

Level	Exercise	Material	Photos	Description
1	Eccentric ER in neutral	Elastic band	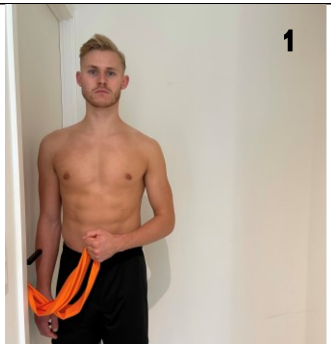 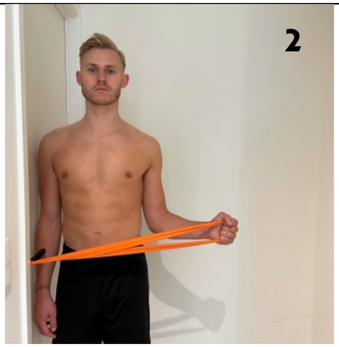 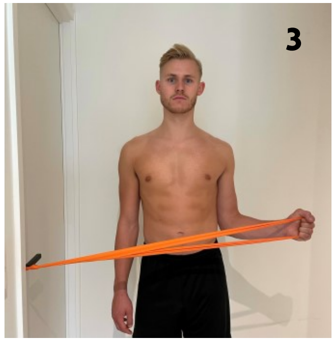 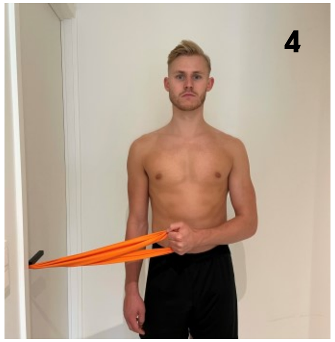	Standing.<br>Stand close to the door with a relaxed elastic band around the door handle as shown in picture 1.<br><br>External rotate the arm as shown in picture 2. Thereby producing tension in the elastic band.<br><br>Take a large step away from the door as shown in picture 3 while keeping the tension.<br><br>Now internal rotate the arm slowly towards the stomach as shown in picture 4 (count to 4).<br><br>Step back towards the door and repeat.<br><br>Variation: Help with the "healthy hand" when external rotating.

2	Eccentric full can	Dumbbell	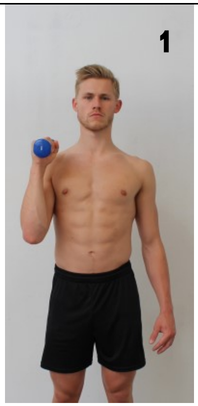	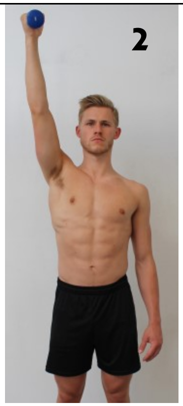	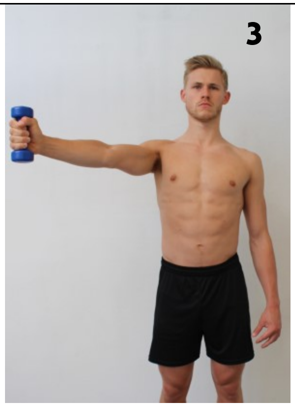	Standing.<br>In part 1 and 2 subject should avoid a concentric elevation, by bringing the hand to the shoulder and then upwards to vertical.<br>The 3rd part is the eccentric part, slowly bringing the arm from vertical to horizontal in the scapular plane.
3	Eccentric horizontal abduction with ER sidelying	Dumbbell	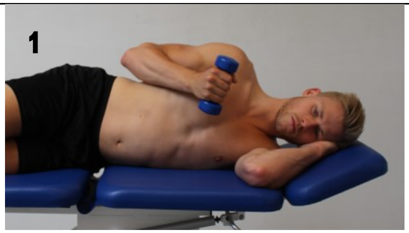<br>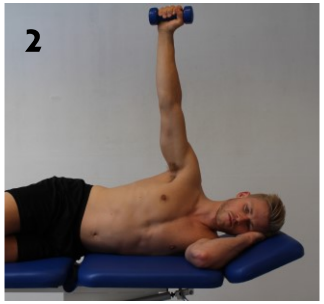<br>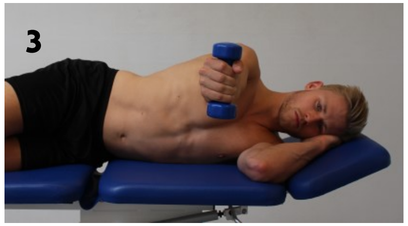			Sidelying.<br>In part 1 and 2 subject should avoid a concentric elevation, by bringing the hand to the shoulder and then upwards to vertical.<br>The 3rd part is the eccentric part, slowly bringing the arm from vertical to horizontal in the frontal plane.

4	Eccentric ER in 90° abduction supine	Elastic band & rolled up towel	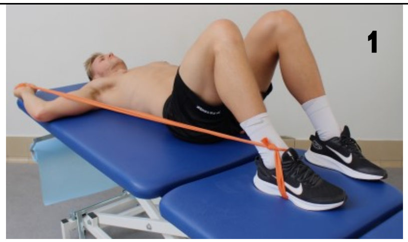<br>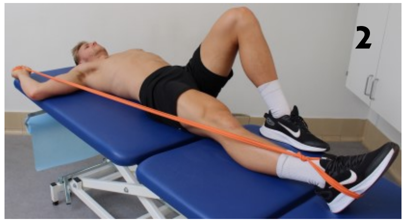<br>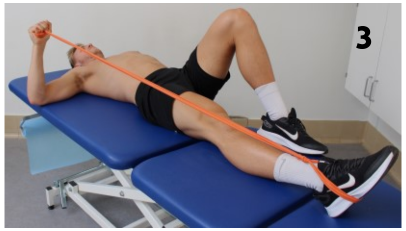	Supine lying.<br>Part 1: the subject should bend the knee<br>Part 2: bring the arm to 90° abduction<br>Part 3: Eccentric part, slowly bringing the arm from horizontal to vertical.<br>If possible, have a rolled-up towel under the upper arm.

PAllow K Isometric exercises

Biomechanical purpose: To perform isometric external rotation and flexion in open kinetic chain.

Generally: Avoid compensating strategies such as shrugging/excessive shoulder elevation, too much flexion in the trunk, head position too much into flexion (protracted) and avoid moving the shoulders forward (protraction) during the exercises.

Purpose from a patient perspective: If any of the above illustrated movements increase pain, isometric exercises may be the choice that induce less pain.

Level	Exercise	Material	Photos	Description
1	External rotation against hand		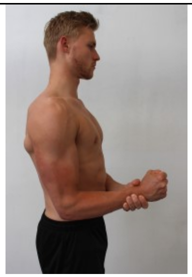	Standing with the side to the wall and elbow flexed 90°. Subjects push the hand against wall or contralateral hand in direction of external rotation.
2	External rotation against towel bilateral	towel	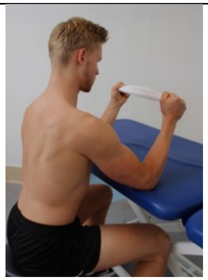	Standing or sitting in front of bench with both shoulders and elbows flexed 90°. Subject hold onto towel and pull in direction of external rotation.
3	External rotation against elastic band	Elastic band	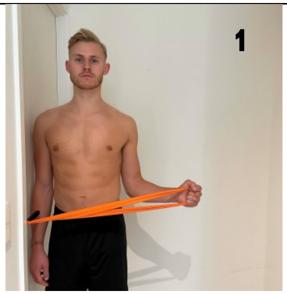 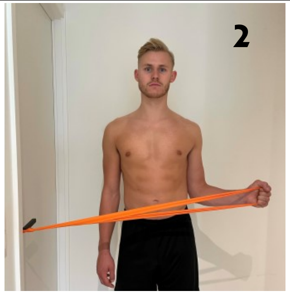	Stand close to the door with the elastic band around the door handle and the arm in external rotation as shown in picture 1. Take a large step away from the door while holding the arm against the stretched elastic band as shown in picture 2. Repetition: 5 of 30-45 sec. x 5 per day
