# Supplementary material for: To allow or avoid pain during shoulder rehabilitation exercises for patients with chronic rotator cuff tendinopathy-Study protocol for a randomized controlled trial (the PASE trial)
Source: Trials. 2024 Feb 21;25:135. doi: 10.1186/s13063-024-07973-6 (PMC10880378; doi:10.1186/s13063-024-07973-6)
Supplement: Supplementary file 4 [file 13063_2024_7973_MOESM4_ESM.pdf]

Kjær et al.
Supplementary file 4

Exercise manual and pain management guidelines for the PAin during Shoulder Exercise trial (the PASE trial)

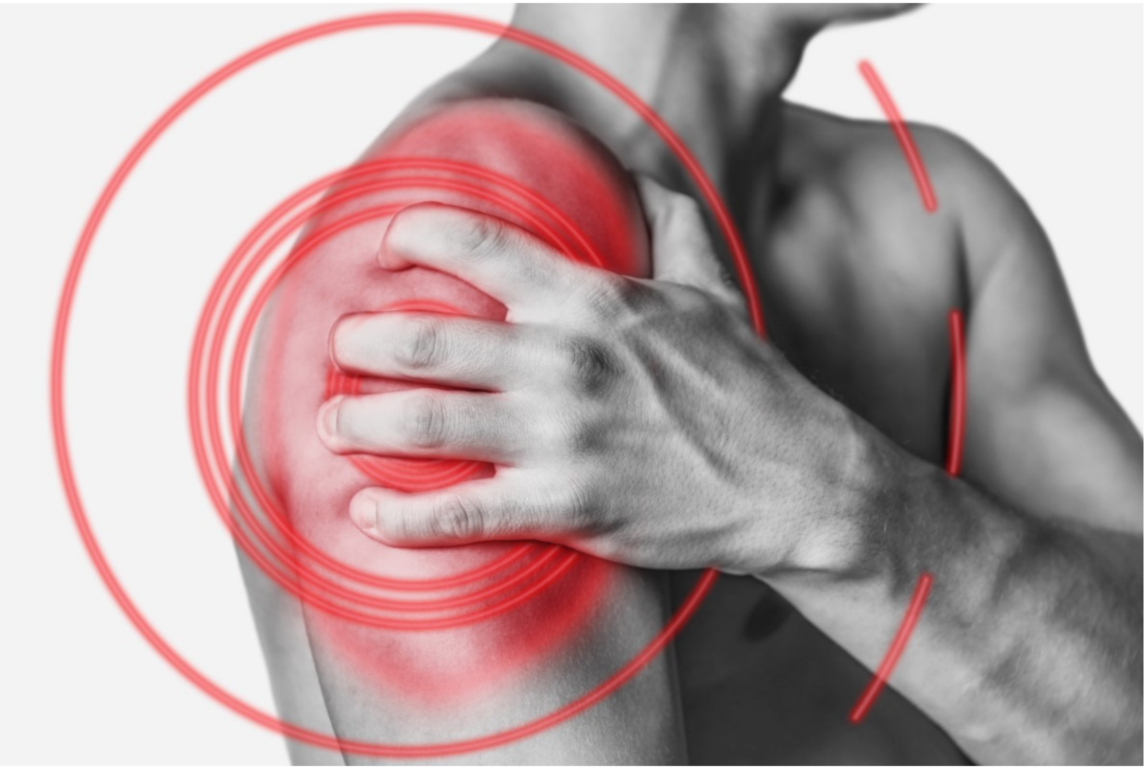

Principal investigator: Birgitte Hougs Kjær Ph.D, Physiotherapist
Department of Physical and Occupational Therapy, Copenhagen University Hospital Bispebjerg and Frederiksberg
Phone: +45 3863 7200, Birgitte.Hougs.Kjaer@regionh.dk

GENERAL INFORMATION ABOUT THE EXERCISE PROGRAMME

1. Management of shoulder symptoms during home exercises
2. Pain monitoring
3. Progression and exercises
4. Other progression parameters

1. Management of shoulder symptoms during exercises

Management of shoulder pain and/or symptoms during exercises is performed with the use of a symptom-scale depicted below. The scale ranges from 0-10, with 10 being worst imaginable symptoms.

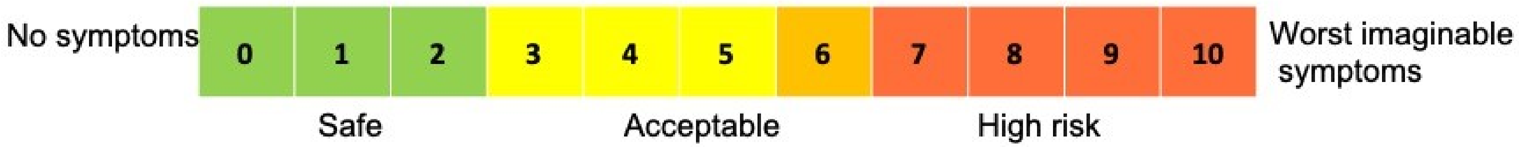

The cutoff regarding progression in relation to pain perception during and after exercises is up to 5/10. However, if you, already before beginning the exercises feel pain of 4/10, the pain is allowed to increase by 2/10 during and after the exercises.

2. Pain monitoring

Self-reported pain levels will be registered in a pain diary.
In connection with a training session, you are asked to report pain level

- before
- during
- immediately after
- 1 hour after
- the morning after

3. Progression and exercises

Exercise F-K are performed in all 26 weeks.

Repetitions: 3 times 15 repetitions with pain as explained above.
How many times a day/week? In general exercises should be performed daily.

- Week 0: Two exercises (1 exercise in the morning and 1 in the evening)
- Week 1: Add 1 exercise = 3 exercises
- Week 4 and on: 4 exercises (varying the exercises from week to week)

4. Other progression parameters

To allow for progression each exercise has between 3 and 18 levels of difficulty.

For all exercises several progression types are possible:

- increasing resistance
- changing the plane
- ball versus towel
- increasing ROM
- increasing kinetic chain demands (perform exercise standing on one leg, or squatting)

An exercise may be progressed when satisfactory neuromuscular control is obtained, according to the following criteria:

- it can be easily performed with good movement quality
- resistance and number of repetitions are achieved
- there are no additional accompanying movements in the body
- it can be performed within the permitted pain limit
- it can be performed with steady breathing and general body control
- It can be performed without need for visual, verbal, or tactile feedback

PAllow F External rotation

Biomechanical purpose: To perform external rotation of the shoulder in open kinetic chain.

Generally: Avoid compensating strategies such as shrugging/excessive shoulder elevation, too much flexion in the trunk, head position too much into flexion (protracted) during the exercises. Also avoid moving the shoulders forward (protraction).
Progression is possible by adding an elastic band.

Purpose from a patient perspective: The exercises in this category are all loading the shoulder tendons. They are important for a good shoulder function, since the shoulder blades and shoulder joints are a constant interplay.

Level	Exercise	Material			Description
1	Side lying external rotation in neutral	Dumbbell	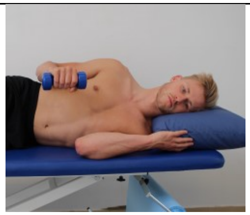	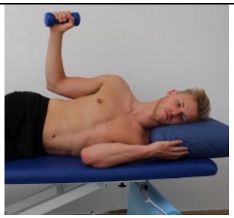	Side lying with rolled up towel or puddle pillow between elbow and trunk.<br>The subject moves the arm (elbow flexed 90°) actively from horizontal to max external rotation and back to the starting position.<br>Progression is possible by adding dumbbell.
2	Standing external rotation in neutral	Elastic band	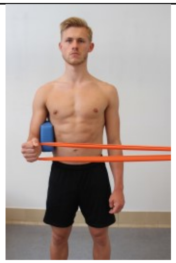	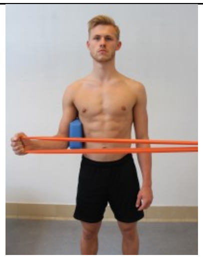	Standing with rolled-up towel or puddle pillow between elbow and trunk. The elastic band is attached to a door handle or similar.<br>Holding the elastic band, the subject moves the arm (elbow flexed 90°) actively from pointing forward to pointing in 45°-60° external rotation and back to the starting position.
3	Sitting external rotation unilateral	Elastic band	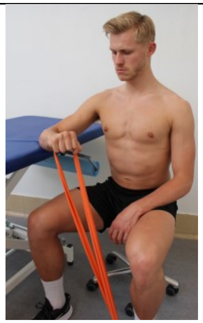	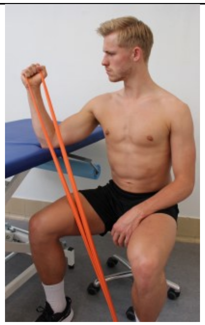	Sitting erect on a chair next to a table/ bench. The elastic band is attached to opposite foot.<br>The elbow is flexed 90° supported by table/ bench and the shoulder is in slight elevation in the scapular plane.<br>Holding the elastic band, the subject moves the arm actively from 45° internal rotation upward to max external rotation and back to the starting position.<br>Further progression is possible by increasing strength of elastic band.

4	Sitting external rotation in loose packed position bilateral (office worker exercise)	Elastic band	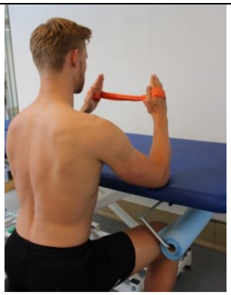	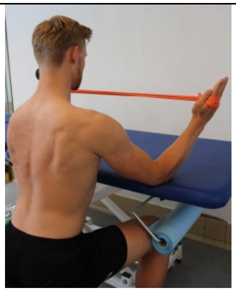	Sitting leaned forward on a chair in front of a table/ bench. The elbow is flexed 90° supported by table/ bench and the shoulder is flexed 30-45°.<br>Holding the elastic band in both hands, the subject moves the arm actively from 30° internal rotation upward to max external rotation and back to the starting position.<br>Further progression is possible by increasing strength of elastic band.
5	Sitting external rotation in 90° abduction	Elastic band	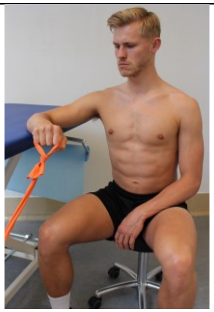	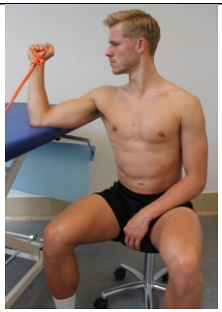	Sitting erect on a chair next to a table/ bench in shoulder level. The arm is resting on the bench with the elbow flexed 90° and shoulder flexed 90°. The elastic band is attached in front of the bench. Holding the elastic band, the subject moves the arm actively from horizontal upward to max external rotation and back to the starting position.<br>Further progression is possible by increasing strength of elastic band.
6	Prone external rotation in 90° abduction	Dumbbell	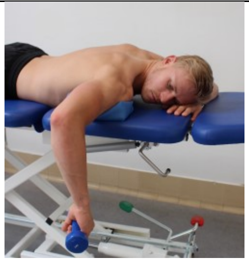	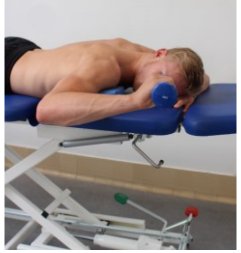	Prone lying on bench with the shoulder/ upper arm resting on a rolled-up towel or puddle pillow with elbow flexed 90° and shoulder flexed 90°.<br>The subject moves the arm actively from vertical upward to max external rotation and back to the starting position.<br>Further progression is possible by adding elastic band.

PAllow G Elevation exercises

Biomechanical purpose: To perform shoulder elevation in open kinetic chain.

Generally:
Avoid compensating strategies such as shrugging/excessive shoulder elevation, too much flexion in the trunk, head position too much into flexion (protracted) during the exercises. Also avoid moving the shoulders forward (protraction).
Progression is possible by increasing weight of dumbbell or exercise weight ball.

Purpose from a patient perspective: The exercises in this category are all loading the shoulder tendons. They are important for a good shoulder function, since the shoulder blades and shoulder joints are a constant interplay.

Level	Exercise	Material	Photos	Description
1	Full can < 90°	Dumbbell	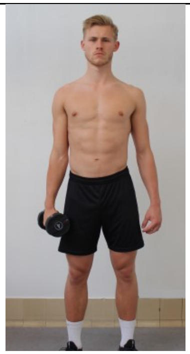 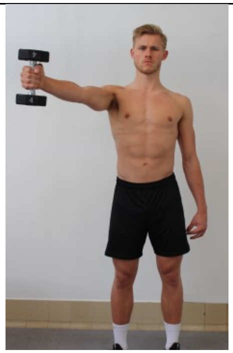	Standing. The subject performs elevation in the scapular plane in open chain to 90° of GH FF and back to the starting position.
2	Full can > 90°	Dumbbell	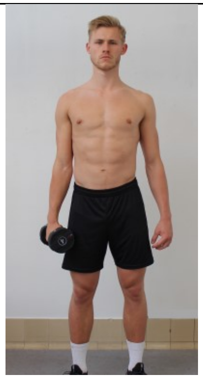 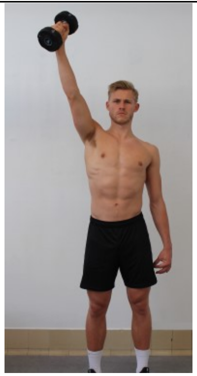	Standing. The subject performs elevation in the scapular plane in open chain to 150° of GH FF and back to the starting position.

3	Prone horizontal abd + ER	Dumbbell	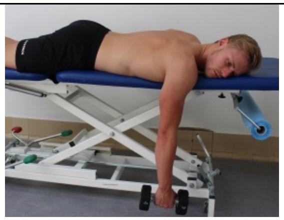 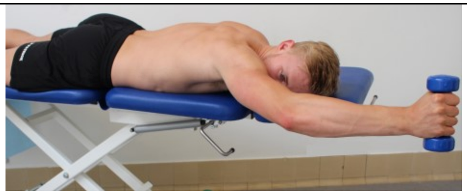	Prone lying. The subject performs a horizontal abduction + ER in open chain to 150° of GH FF and back to the starting position.
4	Prone elevation	Dumbbell	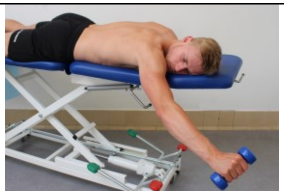 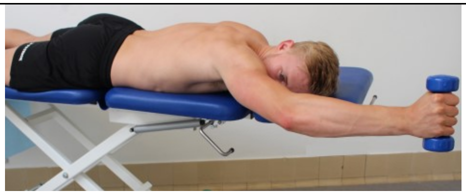	Prone lying. The subject performs elevation in the scapular plane in open chain to 150° of GH FF and back to the starting position.

PAllow H Plyometric exercises

Biomechanical purpose: To perform plyometric exercises by catching an exercise weight ball.

Generally: To add some speed to exercise program.
Progression is possible by increasing weight of exercise weight ball.

Purpose from a patient perspective: To be able to return to sport, work or e.g., gardening it is imperative to add more speed in exercises. The shoulder exercises in this category are all loading the shoulder tendons, however with more speed.

Level	Exercise	Material	Photos	Description
1	Side lying catching in neutral	Exercise weight ball	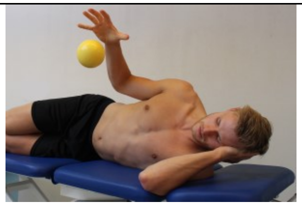 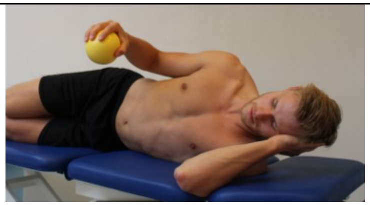	The arm along the side with elbow flexed 90° catching an exercise weight ball.

2	Side lying catching in 90° forward flexion	Exercise weight ball	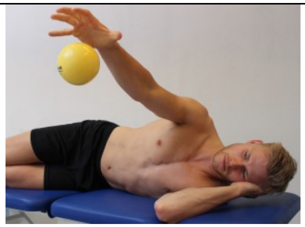	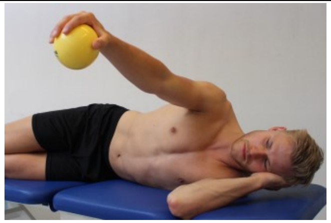	Side lying with shoulder flexed 90° and elbow stretched catching an exercise weight ball.
3	Prone catching 90°-90°	Exercise weight ball	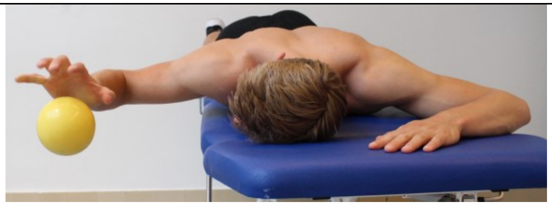 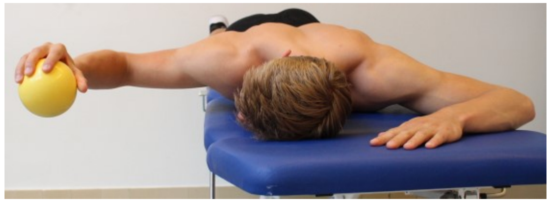		Prone lying with shoulder abducted 90°, ER to horizontal and elbow flexed 90°. Subject is catching an exercise weight ball.
4	Prone catching full elevation	Exercise weight ball	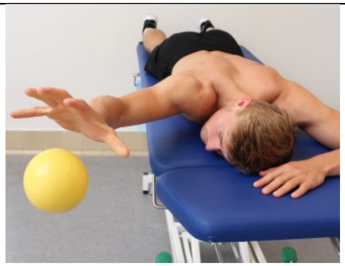	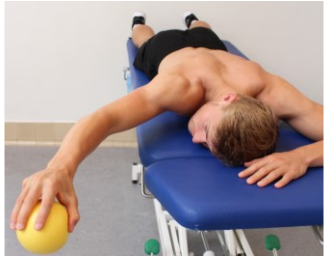	Prone lying with shoulder in full abduction. Subject is catching an exercise weight ball.
5	Standing catching in 90° abduction	Exercise weight ball	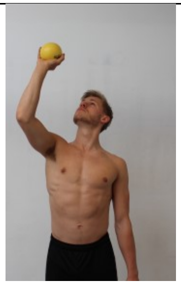		Standing with shoulder abducted 90°, ER to vertical and elbow flexed 90°. Subject is catching an exercise weight ball.

PAllow IJ Eccentric exercises

Biomechanical purpose: To perform eccentric exercises, which means the tendon is loaded with a heavier weight than assumed possible in a concentric way.

Generally: Especially the eccentric part should be performed with good movement quality. Avoid compensating strategies such as shrugging/excessive shoulder elevation, too much flexion in the trunk, head position too much into flexion (protracted) and avoid moving the shoulders forward (protraction) during the exercises.
Progression is possible by increasing weight of dumbbell or exercise weight ball.

Purpose from a patient perspective: In daily life, most of the movement involve tendon loading while the muscle is lengthening (eccentric work). From a functional perspective eccentric work are relevant.

Level	Exercise	Material	Photos	Description
1	Eccentric ER in neutral	Elastic band	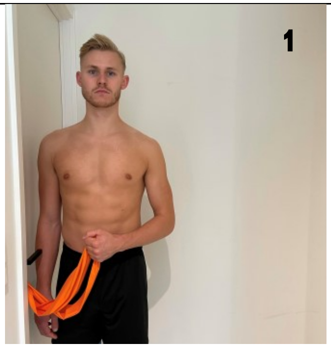 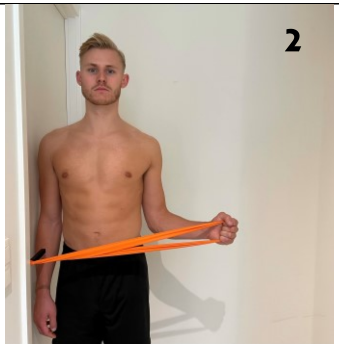 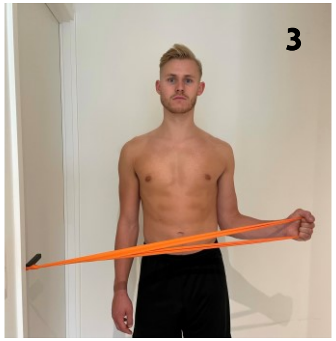 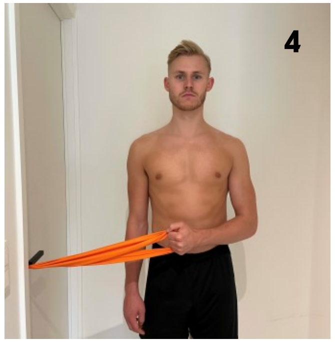	Standing.<br>Stand close to the door with a relaxed elastic band around the door handle as shown in picture 1.<br><br>External rotate the arm as shown in picture 2. Thereby producing tension in the elastic band.<br><br>Take a large step away from the door as shown in picture 3 while keeping the tension.<br><br>Now internal rotate the arm slowly towards the stomach as shown in picture 4 (count to 4).<br><br>Step back towards the door and repeat.<br><br>Variation: Help with the “healthy hand" when external rotating.

2	Eccentric full can	Dumbbell	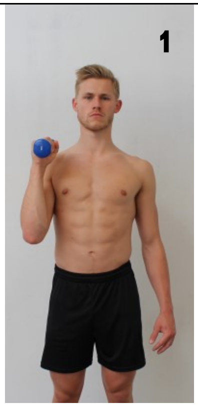	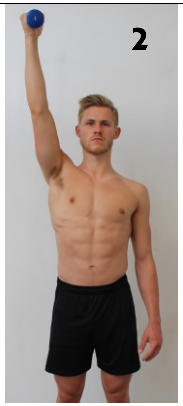	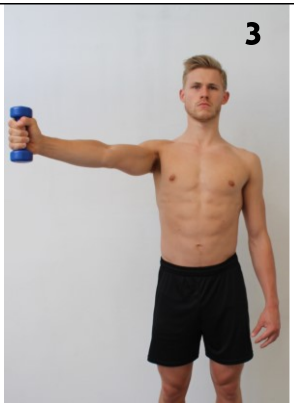	Standing.<br>In part 1 and 2 subject should avoid a concentric elevation, by bringing the hand to the shoulder and then upwards to vertical.<br>The 3rd part is the eccentric part, slowly bringing the arm from vertical to horizontal in the scapular plane.
3	Eccentric horizontal abduction with ER sidelying	Dumbbell	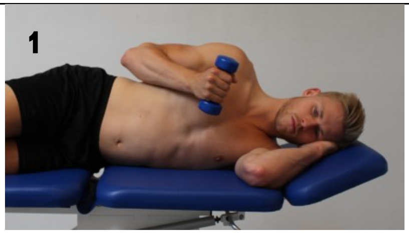<br>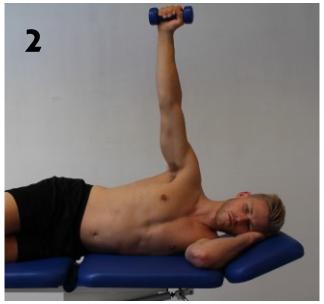<br>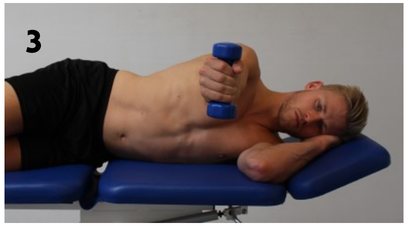			Sidelying.<br>In part 1 and 2 subject should avoid a concentric elevation, by bringing the hand to the shoulder and then upwards to vertical.<br>The 3rd part is the eccentric part, slowly bringing the arm from vertical to horizontal in the frontal plane.

4	Eccentric ER in 90° abduction supine	Elastic band & rolled up towel	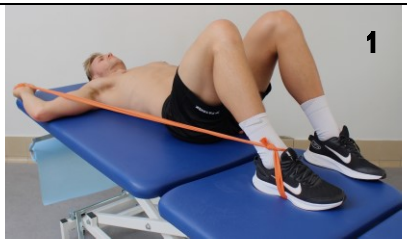<br>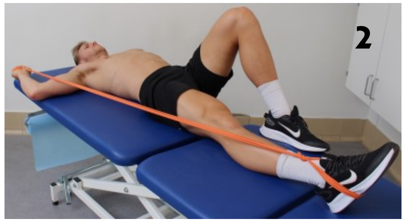<br>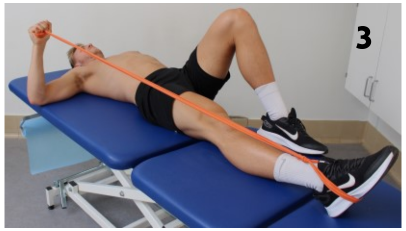	Supine lying.<br>Part 1: the subject should bend the knee<br>Part 2: bring the arm to 90° abduction<br>Part 3: Eccentric part, slowly bringing the arm from horizontal to vertical.<br>If possible, have a rolled-up towel under the upper arm.

PAllow K		Isometric exercises		
**Biomechanical purpose**: To perform isometric external rotation and flexion in open kinetic chain.				
**Generally**: Avoid compensating strategies such as shrugging/excessive shoulder elevation, too much flexion in the trunk, head position too much into flexion (protracted) and avoid moving the shoulders forward (protraction) during the exercises.				
**Purpose from a patient perspective**: If any of the above illustrated movements increase pain, isometric exercises may be the choice that induce less pain.				

Level	Exercise	Material	Photos	Description
1	External rotation against hand		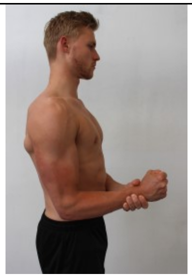	Standing with the side to the wall and elbow flexed 90°. Subjects push the hand against wall or contralateral hand in direction of external rotation.
2	External rotation against towel bilateral	towel	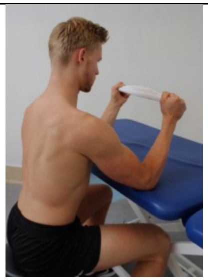	Standing or sitting in front of bench with both shoulders and elbows flexed 90°. Subject hold onto towel and pull in direction of external rotation.
3	External rotation against elastic band	Elastic band	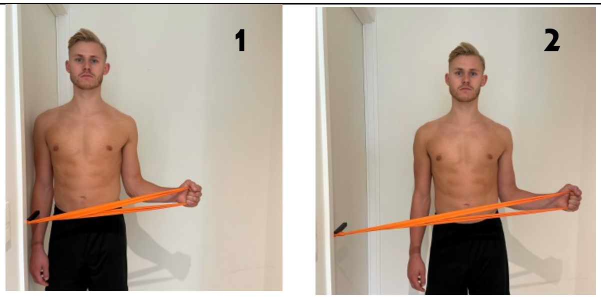	Stand close to the door with the elastic band around the door handle and the arm in external rotation as shown in picture 1.<br><br>Take a large step away from the door while holding the arm against the stretched elastic band as shown in picture 2.<br><br>Repetition: 5 of 30-45 sec. x 5 per day
